# Prime Editing: A Revolutionary Technology for Precise Treatment of Genetic Disorders

**DOI:** 10.1111/cpr.13808

**Published:** 2025-02-27

**Authors:** Mengyao Li, Yi Lin, Qiang Cheng, Tuo Wei

**Affiliations:** ^1^ State Key Laboratory of Stem Cell and Reproductive Biology, Institute of Zoology Chinese Academy of Sciences Beijing China; ^2^ Key Laboratory of Organ Regeneration and Reconstruction, Institute of Zoology Chinese Academy of Sciences Beijing China; ^3^ Beijing Institute for Stem Cell and Regenerative Medicine Beijing China; ^4^ University of Chinese Academy of Sciences Beijing China; ^5^ Department of Biomedical Engineering, College of Future Technology Peking University Beijing China; ^6^ Beijing Advanced Center of RNA Biology Peking University Beijing China

**Keywords:** delivery vehicles, genetic disorders, genome manipulation, precise therapy, prime editing

## Abstract

Genetic diseases have long posed significant challenges, with limited breakthroughs in treatment. Recent advances in gene editing technologies offer new possibilities in gene therapy for the treatment of inherited disorders. However, traditional gene editing methods have limitations that hinder their potential for clinical use, such as limited editing capabilities and the production of unintended byproducts. To overcome these limitations, prime editing (PE) has been developed as a powerful tool for precise and efficient genome modification. In this review, we provide an overview of the latest advancements in PE and its potential applications in the treatment of inherited disorders. Furthermore, we examine the current delivery vehicles employed for delivering PE systems in vitro and in vivo, and analyze their respective benefits and limitations. Ultimately, we discuss the challenges that need to be addressed to fully unlock the potential of PE for the remission or cure of genetic diseases.

## Graphical abstract

This review outlines the latest research advancements in prime editing technology, delivery strategies, and the challenges that must be addressed to fully realize its therapeutic potential, emphasizing the high potential of prime editing in the remission or cure of genetic diseases.

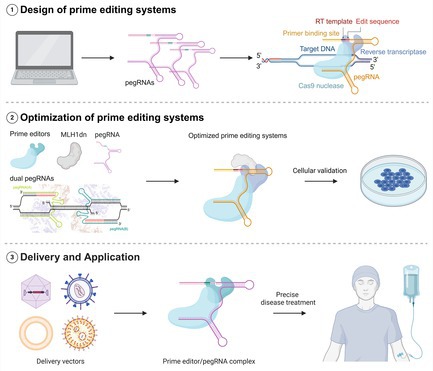

## 1 Introduction

The human genome comprises approximately 21,000 to 23,000 genes that play a critical role in human growth and development [1]. Alterations in these genes can have a profound impact on the overall physical state and physiological function. Human genetic diseases originate from various mutations within the vast genome, including chromosomal disorders, single‐gene disorders (involving one or both alleles), and mitochondrial disorders [2]. To date, numerous human genetic diseases have been identified and extensively studied, leading to an improved understanding of their underlying mechanisms. Various intervention strategies, such as small‐molecule therapy, enzyme replacement therapy, and dietary adjustment, have been developed to alleviate symptoms in diseased individuals [3, 4, 5]. However, these treatments are primarily aimed at symptom relief and cannot fundamentally correct the underlying mutated gene loci, with the result that these mutations are still passed on through inheritance to future generations.

Over the past few decades, significant progress has been made in the field of genetic engineering, which enables the modification of an organism's genetic characteristics [6]. Gene editing therapy, which involves the modification or correction of disease‐causing genetic mutations, has emerged as a promising strategy for treating genetic diseases. Traditional gene editing tools, including zinc finger nucleases (ZFNs) and transcription activator‐like effector nucleases (TALENs), have been extensively used to address these genetic disorders. However, preclinical and clinical studies have revealed certain limitations of these genome editing agents due to the challenges of lack of flexibility, low editing efficiency, and relatively high levels of off‐target effects [7, 8, 9, 10].

**FIGURE 1:**
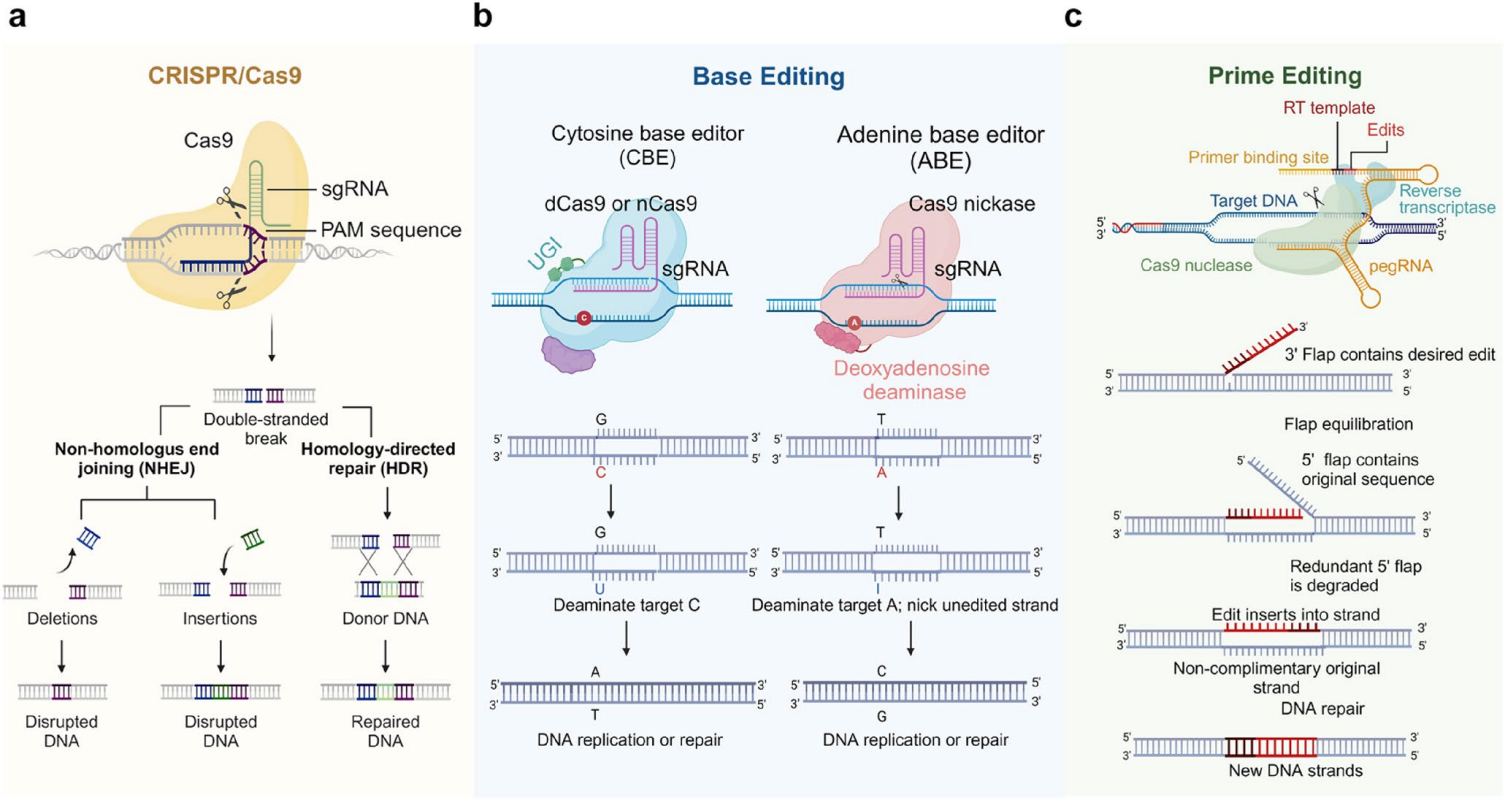
The mechanisms of CRISPR/Cas9 editing, base editing, and prime editing. (a) The CRISPR/Cas9 system usually consists of a Cas9 nuclease and an engineered single‐guide RNA (sgRNA). The sgRNA binds to the Cas9 protein and guides the Cas9 protein to recognize and cleave the target DNA sequence, creating a double‐stranded break. This double‐stranded break is then repaired by intracellular DNA repair mechanisms (NHEJ or HDR), resulting in gene modifications at the repair site. (b) The base editing system typically consists of a base editor protein and a sgRNA. The base editor protein involves the fusion of a catalytically impaired Cas9 enzyme with a cytosine or adenine deaminase enzyme that can convert one DNA base to another (e.g., C‐to‐T or A‐to‐G) at a specific genomic location without causing double‐stranded breaks in the DNA. (c) The prime editing system relies on a fusion of a Cas9 nickase (nCas9) and a reverse transcriptase (RT), together with an engineered sgRNA called prime editing guide RNA (pegRNA). Once the pegRNA recognizes the target sequence, the nCas9 domain of the prime editor nicks one strand of the DNA at a specific position. Next, the RT domain of the prime editor uses the pegRNA as a template to copy and insert the desired new DNA sequence into the nicked site, resulting in the precise modification of the genome DNA. Created with BioRender.com.

The clustered regularly interspaced short palindromic repeat (CRISPR)‐CRISPR‐associated protein 9 (Cas9) system, a powerful genome editing technology that is derived from bacteria, has shown great ability to overcome the above limitations [11, 12, 13]. The CRISPR/Cas9 system consists of two components: a guide RNA (gRNA) and a non‐specific CRISPR‐associated endonuclease Cas9. Once the gRNA recognizes the desired DNA target sequences at the genomic site, the Cas9 nuclease could be activated to induce a double‐stranded break (DSB), which is then followed by repair through non‐homologous end joining (NHEJ) or homology‐directed repair (HDR). NHEJ allows for quick repair of DSBs but may produce random insertions and deletions (indels), while HDR offers the potential for precise editing but is often less efficient and restricted to dividing cells (Figure 1a) [14, 15, 16]. Besides, DSBs may result in substantial translocations, rearrangements, activation of the *p53* gene, and other genomic damages, largely limiting the clinical application of the CRISPR/Cas9 system [17, 18, 19]. To address these challenges, a novel gene editing technology called base editing has been developed, which can achieve precise base substitutions in cellular DNA without inducing DSBs [20, 21]. Till now, adenine base editor (ABE) and cytosine base editor (CBE) are the most commonly used base editors, which enable the conversions of A•T to G•C and C•G to T•A in genomic DNA, respectively (Figure 1b) [20, 21]. It needs to be emphasized that base editors can only work on point mutations and are largely limited to conversions between 4 and 6 single bases. Furthermore, base editors have limitations in their ability to convert or insert and delete other types of bases, as well as perform by‐stander editing. As a result, their application in gene editing therapies is restricted [22].

To overcome these limitations, a groundbreaking gene editing tool called “prime editing” (PE) was developed by Liu and his colleagues in 2019 [23]. PE is considered the most promising gene editing technology, as it enables precise genome modifications in various cell types and organisms with reduced undesired indels at both target and non‐target sites [24, 25]. The PE system consists of two key components: a prime editor protein that integrates a Cas9 nickase (nCas9) and a reverse transcriptase (RT), and a PE guide RNA (pegRNA) that incorporates an additional primer binding sequence (PBS) and a reverse transcription template (RTT) at the 3′ end of gRNA. When the nCas9 is directed to a specific DNA locus by the designed spacer sequence on the pegRNA, it unwinds the double‐stranded DNA and cuts one strand, forming a 3′ flap and a 5′ flap in the nick. Then the PBS hybridizes with the 3′ flap, and the RT synthesizes the desired DNA sequence using the RTT. The excess 5′ flap lacking the desired edit is degraded by structure‐specific endonucleases in the cellular DNA repair process, while the new 3′ flap is ligated to form an edited DNA strand. The resulting heteroduplex of the edited and unedited DNA strands can be resolved by DNA repair, permanently integrating the target edit (Figure 1c). Since its appearance, PE has been applied in diverse organisms, including plants, zebrafishes, human embryos, and human induced pluripotent cells [26, 27, 28]. Currently, several preclinical studies on PE based gene editing therapies are ongoing (Table 1), but the whole research field of PE is still in the preliminary research stage and requires continuous optimization to solve its current problems, such as low editing efficiency, large size, complex design, and off‐target effects. In this review, we first describe the development of canonical PE systems and their variants with improved performance or specific functions. Then, we summarize the delivery techniques used in vitro and in vivo for PE systems. Finally, we discuss the current challenges and future directions of PE based therapeutic applications. We believe that, by overcoming these challenges, PE holds the promise of fully unlocking the potential of diverse gene editing applications.

**TABLE 1:** The applications of PE in preclinical gene therapies.

Mutation type	Disease	Molecular mechanism	PE system	Efficiency	Delivery method	Reference
Single base replacement	Phenylketonuria	*Pah* gene C to T conversion	PE2	11.1% ± 3.3%	Human adenoviral vector 5	[29]
	Alpha‐1 antitrypsin deficiency	*SERPINA1* gene G to A conversion	PE2*	6.7%	Hydrodynamic injection	[30]
	Sickle cell disease	*HBB* gene A to T transversion	PE3	58%	Lipofectamine 2000	[23]
	Increase resistance to prion disease	*PRNP* gene G to T transversion	PE3	53%	Lipofectamine 2000	[23]
	Hereditary tyrosinemia type 1	*Fah* gene G to A conversion	PE3 or PE3b	2.3%	Electroporation	[31]
	Beta‐thalassemia	*HBB* gene C to T conversion	PE3	14.29%	Microinjection	[32]
	Leber congenital amaurosis	*Rpe65* gene C to T conversion	PE2	6.4%	Adeno‐associated virus vector	[33]
	Recessive dystrophic epidermolysis bullosa	*COL7A1* gene T‐to‐C and T‐to‐G conversion in two sites	PE3	10.5%	Electroporation	[34]
Deletion/ insertion of small fragments	Duchenne muscular dystrophy	Mutations impairing RNA splicing or translation of *DMD* gene	WT‐PE	~6.8%	Transeasy	[35]
	Tay–Sachs disease	*HEXA* gene contains a 4‐bp insertion	PE3 and PE3b	> 20%	Lipofectamine 2000	[23]
	DGAT1‐deficiency	3‐bp deletion in exon7 of *DGAT1* gene	PE3	21%	Electroporation	[36]
	Cystic Fibrosis	*CFTR* gene contains a 3‐bp deletion	PE3	> 20%	Electroporation	[37]
	Spinal muscular atrophy	The lack of *SMN2* gene exon7	PE3	18.61%	jetPRIME	[38]
Recoding large fragments	Phenylketonuria	The exon 4 and exon 7 of *PAH* recoding	Twin‐PE	23% for a 46‐bp recoding in exon 7; 27% for a 64‐bp recoding in exon 7; 9.4% for a 64‐bp recoding in exon 4	Lipofectamine 2000	[39]
Other changes of genome	Hunter syndrome	40‐bp inversion between *IDS* and *IDS2*	Twin‐PE	9.6%	Lipofectamine 2000	[39]

## 2 Development of PE Systems

Efficient and precise intracellular PE relies on several crucial factors, including the design of pegRNA, maintenance of pegRNA integrity, suitable delivery tools, successful assembly of the PE‐pegRNA ribonucleoproteins (RNPs), effective target recognition by the RNP complexes, efficient reverse transcription, and minimal production of unintended byproducts. To enhance the performance of PE, Liu and co‐workers made important modifications based on their initially designed PE systems to improve their efficiency and minimize undesired editing outcomes. Additionally, other researchers have also optimized the PE systems in various aspects, and all of these variants are expected to broaden the application scope and improve the editing efficiency of PE editing, thereby paving the way for future fundamental research and therapeutic applications in the clinic.

### 2.1 Development of Original PE Systems

#### 2.1.1 PE1 and PE2

PE1 is the original version of prime editors, which includes a fusion protein that combines the *Streptococcus pyogenes* Cas9 nickase (SpCas9, H840A mutant) and the wild‐type RT domain from Moloney murine leukemia virus (M‐MLV) as the editor protein, and a specific pegRNA to specify the target genomic site [23]. The editing efficiency of PE1 for introducing all 12 types of single‐base substitutions and small insertions and deletions at the HEK3 locus was validated in human cells, suggesting the expanded scope and capabilities of PE. However, the overall editing efficiency of PE1 was modest, with less than 6% for point mutations and 17% for targeted insertions and deletions. To overcome this limitation, the second‐generation prime editor, PE2, has been developed. PE2 incorporates five mutations into the wild‐type RT domain of PE1 to improve its thermostability, enzymatic processivity, and target DNA binding affinity for PBS of pegRNA. As a result, PE2 exhibited a significant improvement in editing efficiency, achieving 1.6‐ to 5.1‐fold enhancement with < 0.5% of off‐targets in the point mutations [23].

#### 2.1.2 PE3 and PE3b

Once the desired edit has been incorporated into the 3′ flap and the 5′ flap has been excised by flap interconversion, there is a risk that the cellular mismatch repair (MMR) mechanism could revert the edited heteroduplex DNA back to its original state, significantly reducing the editing efficiency of prime editors [40]. To address this issue, a strategy inspired by previous base editors has been introduced that uses an additional single guide RNA (sgRNA) to create a nick in the non‐edited strand. This approach generates the PE3 system, which can induce two lesions around the target site and force the eukaryotic MMR system to repair the additional nick using the desired edited strand as a template. The PE3 substantially improved the editing efficiency by up to 55% and 1.5‐ to 4.2‐fold enhancement at four loci compared to PE2. However, the coincident presence of simultaneous two nicks on both DNA strands could result in the DSBs, leading to an increased formation of editing byproducts such as indels [23, 41]. To minimize the generation of DSBs and indels, Anzalone and colleagues developed the PE3b system by introducing a modification to the PE3, in which a special sgRNA targeting the edited sequence was incorporated to nick the non‐edited strand. The PE3b system significantly reduces the frequency of indels, although its efficiency may vary in different cell types [23]. Overall, the inclusion of a nicking sgRNA has been shown to substantially improve the editing efficiency of PE editing, making it a promising technique for future fundamental research and therapeutic applications [30, 31].

#### 2.1.3 PE4 and PE5

To further increase the editing efficacy of prime editors, Chen et al. attempted to attenuate the effect of the MMR process, for example, by silencing the MMR gene with siRNA, and observed increased editing efficiency accompanied by a reduction of editing byproducts [40]. This finding suggests that the MMR mechanism may identify and fix the modifications made to the target site during PE, resulting in a decrease in editing efficiency. Based on it, PE2, pegRNA, and various dominant negative MMR protein (MLH1dn) variants were co‐transfected into HEK293T cells to assess the efficiency and precision of PE editing. In MMR‐proficient cells, MLH1dn inhibited MMR and greatly increased the editing efficiency in a dose‐dependent manner, while no improvement was observed in MMR‐deficient cells such as HCT116 cells [42]. The produced system, termed PE4, which contains the PE2 system and MLH1dn, demonstrated a 6.5‐fold improvement in editing compared to the original PE2. Similarly, the PE5 system, consisting of the PE3 system and MLH1dn, exhibited a 1.9‐fold enhancement in editing efficiency compared to the initial PE3, with improved editing purity and reduced unintended editing byproducts at multiple loci in human MMR‐proficient cells. The improvement also occurred in MMR‐deficient cells, although the improvement was less than that in MMR‐proficient cells. All of these results indicate that PE4 and PE5 systems consisting of the MLH1dn domain can remarkably increase the efficiency of PE editing with fewer byproducts. However, further safety evaluation of PE4 and PE5 is required due to the potential risk of genomic mutations induced by MMR interference [40].

#### 2.1.4 PE6a‐g

In order to make the PE editing system smaller and enable more efficient delivery through vectors such as AAV, scientists are dedicated to reducing the size of the RTs. Doman et al. compared 59 RTs from 14 different classes in HEK293T cells. The results showed that some of these RTs are smaller than M‐MLV RT but are less efficient. The researchers then attempted to overcome the low editing efficiency with protein engineering. They used a phage‐assisted continuous evolution (PACE) approach. The efficiency of these RTs as primer editors was then evaluated in HEK293T cells. The results showed that the editing efficiency of the evolved RTs greatly exceeded that of their wild‐type RT counterparts. Among them, evo‐Ec48 is the most compact RT (1.2 kb), and the prime editor derived from it is named PE6a. evo‐Tf1 exhibits an editing efficiency comparable to PEmax, and the prime editor derived from the evo‐Tf1 RT is named PE6b. When testing the editing efficiency of PE6a, PE6b and PEmax on the Tay‐Sachs cell model under the addition of nicking sgRNA, the average *HEXA* correction rates were 16%, 53%, and 46%, respectively. However, the current PE6a and PE6b are typically less efficient for long, complex edits. Further evolution to support long and complex editing followed. Evolved Tf1 variants (PE6c) and engineered M‐MLV variants (PE6d) were generated, both of which were more efficient at editing large fragments and TWIN‐PE.

It was found that some conserved mutations in the Cas9 structural domain were also acquired during the successive evolution of prime editor. Combinations of these mutations were tested to identify the best‐performing engineered Cas9 variant, termed PE6e‐g. The PE6e‐g variants improved the efficiency of PE in some edits, but most of the mutations reduced editing efficiency, possibly due to the reduced affinity of the mutant Cas9 for DNA. The PE6 editor demonstrated minimal off‐target effects in gene editing both in vitro and in vivo, indicating a robust safety profile. Its compact size and efficient efficiency enable its application in animal models and ultimately in human patient therapy [43].

#### 2.1.5 PE7

By conducting in‐depth research on the interactions between key components of PE technology and the cellular environment, Yan et al. conducted a genome‐scale CRISPRi screen, identifying a crucial component of PE: the short RNA‐binding exonuclease protection factor (La). Increased expression of La could improve the overall editing efficiency in cells. The possible reason may be that La enhances the stability and integrity of pegRNAs and epegRNAs by interacting with their 3′ ends. The La (1–194) was then fused to the C‐terminal of PEmax (called PE7), which showed enhanced editing efficiency compared with PEmax at eight genomic loci in the U2OS cell line, with negligible effects on off‐target editing. Simultaneously, its editing efficiency at several disease‐related targets was enhanced by over tenfold compared to the current PEmax, providing renewed optimism for the treatment of genetic disorders [44].

### 2.2 Strategies for Improved PE Performance

#### 2.2.1 Optimization of Prime Editor Protein

Efficient nuclear entry of genome editing agents is a crucial step for effective cellular gene manipulation. Studies have shown that optimization of the nuclear localization signal (NLS) sequence can affect the efficacy of gene editing [45, 46]. To this end, Liu et al. developed a PE2* system by adding a c‐Myc NLS at the N‐terminus as well as fusing a variant bipartite SV40 NLS with SV40 NLS at the C‐terminus of the PE protein [30]. The PE2* system exhibited improved nuclear localization and enhanced activity in base conversions and small insertions and deletions compared to the original PE2 system. Using a similar strategy, Chen et al. also made modifications to prime editors by adding the bipartite SV40 NLS to the linker region between the RT and Cas9 domains and the c‐Myc NLS to the C‐terminus of the PE protein, achieving significantly increased editing frequency compared to the original PE architecture [40]. The optimization of the NLS was also utilized by Chen et al. to develop another optimized version of the PE2 protein, called PEmax. They added the bipartite SV40 NLS and a c‐Myc NLS to the C‐terminus of the PE2 protein. The codons of RT and the nCas9 mutation were also optimized to obtain the final PEmax. In HeLa cells, the PE2max, PE3max, PE4max, and PE5max systems using the PEmax architecture increased the expected editing frequency on average [40]. These findings highlight the importance of NLS composition and architecture in enhancing the efficacy of PE systems.

Beside insufficient nuclear entry, Velimirovic et al. found that the limited interaction strength between PE systems and target sites is one of the reasons contributing to the low efficiency of PE editing [47]. To solve this restriction, peptides were tethered to PE2 to enhance the expression of prime editor proteins. Speculating that peptides from DNA repair proteins might encode structural domains capable of altering the efficiency of PE, they constructed peptide libraries by choosing 12,000 peptides consisting of 85 amino acids from DNA repair proteins. The peptide candidates have been integrated into the PE2 editor using a 33‐amino acid XTEN linker for high‐throughput screening. In particular, the dual peptide‐PE2 system, named IN‐PE2, exhibited the highest PE editing efficiency across different cell lines. The underlying mechanism might be that these peptides increase the transcription and translation efficiency of the PE2 enzyme. In addition to stabilizing the prime editor proteins, the ssDNA binding protein domain (ssDBD) was incorporated to stabilize the single‐stranded DNA (ssDNA) formed between the pegRNA and the target DNA sequence. Song et al. added DNA binding domains (DBDs) to the prime editor proteins using linkers with different lengths and investigated their editing efficiency [48]. By adjusting the types of the DBDs, the length of the linkers, and the location of the DBDs (N‐ or C‐terminal regions of PE2), they identified the Rad51 DBD as a suitable candidate. The Rad51 DBD was previously reported to bind to both ssDNA and RNA, which promotes double‐strand hybridization [49]. This modified system, named hyPE, achieved an average 2.0‐fold editing enhancement in primary human skin fibroblasts. Similar improvements were also achieved in other cells, although a slight increase in byproduct formation was observed.

In addition, Zong et al. engineered the plant prime editor (ePPE) by removing the ribonuclease H (RNase H) domain and inserting a viral nucleocapsid protein with nucleic acid chaperone activity [50]. The resulting prime editor with two modifications synergistically increased the efficiency of PE editing by 5.8‐fold at multiple genomic sites in plant cells, with minimal editing byproducts detected.

#### 2.2.2 Optimization of pegRNA

##### 2.2.2.1 Engineering of 3′ Extension

RNA is known to be inherently susceptible to degradation due to its unique structure and the presence of endogenous or exogenous RNases in the environment or in cells. Regarding the PE system, the exposed PBS and RTT regions of the pegRNA are vulnerable to being degraded by RNases, resulting in truncated pegRNAs. Additionally, the unstructured 3′ extension of the pegRNA may further destabilize the structure, which hinders the efficiency of PE by introducing competition between full‐length pegRNA and truncated pegRNA for target sites. Therefore, improving the stability of pegRNAs is crucial to enhancing the editing efficacy. To this end, Zhang et al. introduced viral exoribonuclease‐resistant RNA motifs (xrRNAs) into the 3′ end of pegRNAs to protect them from exonuclease degradation [51, 52]. The inclusion of xrRNA motifs significantly increased the editing efficiency of PE2 and PE3 at various genomic loci in multiple cells. When xrRNA was introduced into the PE3 framework, the editing effectiveness of xrPE was 9.1 times higher than canonical PE3 at six insertion or deletion sites in N2A cells. In HEK293T cells, the editing efficiency of xrPE was 5.5 times higher than canonical PE3 at the6 individual sites. Importantly, xrPE retained the precise targeting specificity of PE3 and exhibited similar performance to PE3 in terms of insertion or deletion ratios and minimal off‐target editing [52]. Nelson et al. reported a similar strategy to engineer pegRNA (called epegRNA) by incorporating a structured RNA motif (tevopreQ1 or frameshifting pseudoknot) at the 3′ end. This modification prevented the degradation of the 3′ end of the pegRNA, improved editing efficiency by 3‐4‐fold in various types of mammalian cells, and maintained minimal off‐target effects and editing byproducts [53].

Using a similar strategy, Feng et al. appended a 3′‐stem‐loop aptamer (MS2) to the 3′ end of pegRNA to prepare the stem‐loop PEs (sPE), which enhanced the stability of PE2‐pegRNA complexes [54]. Consequently, the editing efficiency of the sPE showed an average improvement of 1.8‐fold in HEK‐293 T and other cells. The addition of the G‐quadruplex motif to the 3′ extension of the pegRNA was also tested, where a maximum editing efficiency of over 80% at endogenous loci was achieved [55]. Overall, the fusion of different types of RNA motifs into pegRNAs could preserve the integrity of the 3′ end and provide a universal approach to improving editing efficacy, but it also may complicate the production of pegRNAs due to the increased RNA size.

In addition to the degradation, the pegRNA circulation caused by the self‐annealing between the partially complementary sequences of the 3′ and 5′ ends can potentially hamper the editing outcomes. To solve this problem, Liu et al. fused the 20‐nt Csy4 recognition site to the 3′ end of the pegRNA to prevent the circularization by forming a hairpin structure [56]. In order to simplify the calculation, the nick‐sgRNA was combined with the extended pegRNA so that they could be expressed in a single transcript. Simultaneously, the Csy4 RNase was fused to the PE editor to specifically cut the 3′ end of the Csy4 recognition site to release the nick‐sgRNA. This combination strategy significantly boosted the editing efficiency of the PE system, although it also resulted in a slightly higher ratio of indels.

##### 2.2.2.2 Optimization of pegRNA Sequence

The above sections have discussed that the effectiveness and precision of PE editing are adversely affected by the MMR. Notably, multi‐point mutation editing has been found to circumvent the MMR repair pathway. It has also been observed that the MMR pathway exhibits low sensitivity towards single‐base mutations and is particularly less proficient in detecting G‐to‐C mutations. Based on these findings, the efficiency of PE editing can potentially be improved by introducing silent or benign mutations near the desired editing site to generate a mutant that is relatively insensitive to the MMR. Li et al. developed two such pegRNAs for the PE3 and PE5 systems [57]. Same‐sense mutations were introduced into the RTT region of pegRNA (referred to as spegRNA), and the optimal one produced 353‐fold higher editing efficiency compared to pegRNA. The pattern of introducing such mutations revealed that less than four base substitutions in the RTT region significantly improved editing results. Additionally, a new pegRNA called apegRNA was constructed, in which a C/G pair is inserted or each non‐C/G pair is modified into a C/G pair in the small hairpin of pegRNA, eventually altering the sequence of pegRNA to stabilize its secondary structure. Given that the modification of these two pegRNAs affects different parts of the pegRNA, the authors explored the combination of the aforementioned two modifications to enhance editing efficiency. The combination of spegRNA and apegRNA in the PE3 and PE5 systems led to a significant improvement in editing efficiency at multiple gene loci while maintaining comparable editing fidelity. Similar strategies have been adopted by other research groups to optimize pegRNA sequences and improve PE efficiency. For instance, the RTT region of pegRNA was modified by introducing the protospacer adjacent motif sequence and a silent mutation [58]. This modification improved the point correction (approximately 22% modification) in the DMD gene in patient myoblasts.

Intra‐molecular and inter‐molecular auto‐inhibitory interactions of pegRNA can also negatively impact its assembly with prime editor proteins, thereby potentially reducing the editing efficiency [59, 60]. Ponnienselvan et al. and Zheng et al. found that reducing the complementarity of the 3′ and 5′ regions of pegRNAs was able to increase PE efficiency with multiple PE formats [59, 60]. In addition, a shorter PBS region and a melting temperature of the PBS‐DNA target site near 37°C were found to be optimal in mammalian cells [59]. Interestingly, another independent study reported that the optimal melting temperature of PBS was 30°C in rice [26].

##### 2.2.2.3 Split or Paired pegRNAs

As mentioned above, the production of long pegRNAs with additional 3′ modifications may pose a challenge. To tackle this problem, Feng et al. created the split pegRNA prime editor (SnPE) by dividing the canonical pegRNA into two parts: the sgRNA and prime RNA (pRNA), where pRNA binds to the N‐terminal RNA binding proteins (RBPs) of the engineered RBP‐Cas9 nickase‐MMLV [54]. The SnPE showed comparable editing efficiency with the canonical PE systems in mammalian cells and increased the flexibility of PE editing. Using a different strategy, Lin et al. employed two separate pegRNAs (NGG‐pegRNA and CNN‐pegRNA) in trans encoding the same edits for the forward and reverse strands simultaneously [26]. They found that dual pegRNAs substantially improved PE efficiency, with 4.2‐fold and 1.8‐fold higher on average than individual NGG‐pegRNA and CNN‐pegRNA, respectively.

#### 2.2.3 Co‐Administration With Other Agents

Previous studies have shown that p53‐dependent responses can impede gene editing in various cell types. To investigate whether transient inhibition of p53 could improve the editing efficiency of PE editing, Li et al. co‐electroporated the PE2 and PE3 systems with a p53 carboxyl‐terminal dominant‐negative (P53DD) vector in a GFP reporter cell line [61]. Remarkably, a significant improvement in GFP expression (2‐ to 3‐fold) was observed 24 h post‐electroporation. Furthermore, the addition of P53DD resulted in a 3‐ to 4‐fold improvement in editing efficiency at specific endogenous loci or disease‐relevant mutation loci in human pluripotent stem cells (hPSCs), with a moderately increased frequency of indels compared to the PE system alone. It is important to note that other p53 inhibition agents, such as p53 siRNA or small‐molecule inhibitors, failed to enhance the desired editing efficiency, likely due to their differences in kinetics.

In an independent study, Liu et al. conducted a screening of small molecules that might affect the editing efficiency of prime editors in HEK293T cells [62]. A group of HDAC inhibitors (HDACi) was identified to remarkably improve the efficiency of insertions and deletions of small fragments (40 bp) but reduce the editing frequency of point mutations. HDAC inhibitors are thought to promote an open chromatin state by catalyzing the acetylation of histones or other non‐histone proteins, thereby facilitating the engagement of prime editors at target sites. However, further investigations are still required to elucidate the underlying mechanisms of their role in PE editing. Ferreira da Silva et al. found that MMR factors such as MLH1 are recruited to PE editing sites and inhibit the editing process [63]. By transiently depleting MLH1 with a pool of siRNAs, an approximately 2‐fold increase in editing efficiency was achieved at the HEK3 locus in HEK293 cells.

#### 2.2.4 Combination of Multiple Strategies

To maximize PE editing efficiency in certain applications, the combination of multiple strategies to modulate both exogenous prime editors and endogenous pathways is necessary. For example, Qi et al. used triple strategies, including pegRNA optimization, reverse transcription modulation, and HDAC inhibitor administration, to enhance the PE efficiency in the porcine genome [64]. The synergistic combination strategy demonstrated dramatically enhanced PE editing at multiple genomic sites in porcine embryonic fibroblasts (PEFs), providing a practical approach to modifying the pig genome. Besides, Park et al. developed a CMP‐PE system using proximal dead sgRNA (dsgRNA) and chromatin‐modulating peptides (CMPs) to increase PE efficiency at various target sites in mouse cells and embryos [65]. The dsgRNA is the engineered sgRNA with a reduced RNA targeting sequence length of 14–15 nucleotides (nt) and the addition of MS2 binding loops. The dsgRNAs are able to unwind the chromatin structure of the target sites and mediate robust gene activation by means of an active Cas9, thus controlling chromatin close to the target site [66]. CMPs consisting of high mobility group nucleosome binding domain 1 (HN1) and histone H1 central globular domain (H1G) were fused to nCas9 to increase editing efficiency through their natural interaction with chromatin [67]. Using this combination strategy, the efficacy of PE was increased up to 4‐fold for single‐base conversions and insertions of one or more nucleotides compared to the conventional PE system.

In a recent study, Liu et al. enhanced the editing activity of MMLV‐RT‐based PE through improvements in PE solubility, MMLV‐RT dNTP affinity, and intracellular dNTP availability. The improved solubility of the prime editor (called PEmax**) was achieved by the combination of V223M and L435K MMLV‐RT mutations. The generated PEmax** exhibited significantly higher PE editing efficiency than PEmax by an average of ~2.5‐fold and displayed a modest improvement over PE6d by approximately 1.4‐fold across the panel of target sites. In addition, when co‐delivering an accessory protein VPX that degrades SAMHD1 to increase intracellular dNTPs or supplementing cellular growth medium with dNTPs, the PE rates of the PEmax** could be further increased. The developed combination strategies were envisioned to be used with in vivo delivery platforms, such as lipid nanoparticles (LNPs) and virus‐like particles (VLPs), and potentially improve PE editing outcomes in organ systems with low dNTP levels [68].

#### 2.2.5 Optimization of pegRNA Synthesis

pegRNA, especially the engineered epegRNA, is longer than sgRNA. The synthesis of pegRNA typically uses solid‐phase chemical synthesis, but this method has low yield and poor product quality. This leads to suboptimal editing efficiency when using ribonucleoprotein (RNP) and RNA delivery. Lei et al. devised a quick, efficient, and economical technique for producing chemically modified pegRNA (125–145 nt) and epegRNA (170–190 nt) to improve the editing efficacy of PE. The researchers employed a technique for RNA synthesis called splint ligation. Through the optimization of splint ligation parameters, including ligation temperature, splint DNA length, RNA to splint DNA ratio, and ligase dose, they achieved approximately 90% production efficiency for these RNAs. The generated epegRNA (170–190 nt), designated as L‐epegRNA, incorporates modified nucleotides at both the 5′ and 3′ end and contains an evopreQ1 motif at the 3′ end. L‐epegRNA has exhibited enhanced editing efficiency across multiple cell lines and human primary cells. It increased the editing efficiency by more than tenfold with RNP delivery and hundreds of times with RNA delivery compared to epegRNA produced by in vitro transcription (IVT). Furthermore, L‐epegRNA shows significant potential for the insertion of long segments. This technology has facilitated the advancement of PE [69].

### 2.3 Prime Editor‐Derived Variants

#### 2.3.1 Novel PE Variants Designed to Overcome Targeting Limitation

Conventional PE systems are limited by the available PAM sequences and the distance over which editing can occur. *Streptococcus pyogenes* Cas9 (spCas9) protein is used in canonical PE systems, which predominantly recognizes the NGG PAM sequence, restricting the range of PAM options. To expand the editing possibilities, researchers have explored the use of PAM‐flexible Cas9 variants with different nicking capabilities. Efforts to modify PAM recognition typically involve the exploration of natural spCas9 homologues and the manipulation of PAM recognition using protein engineering techniques.

Researchers identified different PAM sequences recognized by various Cas9 variants, including NGA [70], NGCG [70], NG [71], and NRN [72]. Based on these findings, Kweon et al. engineered each variant to generate its nickase by introducing the H840A mutation and fused these variants to the RT domain to generate different PE2 systems (including PE2‐VQR, PE2‐VRQR, PE2‐VRER, PE2‐NG, PE2‐SpG, and PE2‐SpRY) with a broader range of PAM recognition [73]. These PE2 variants were compared in correcting various types of mutations, for example, the BRAF V600E mutation that involves a T:A to A:T conversion, which is one of the most common clinical mutations in cancer [74]. In this mutation, the pegRNA nick site is 18 nt from the target site, rendering the standard PE system ineffective. The results showed that PE2‐SpRY improved the editing efficiency by 6.7‐fold (from 0.3% to 1.8%), and the efficiency was further increased to 11.8% by using PE3b systems. Liu et al. utilized a *Staphylococcus aureus* Cas9 (SaCas9) nickase (N580A) to replace the SpCas9 nickase, expanding the range of PAM sequences while maintaining comparable editing efficiency [30]. Besides, Oh et al. replaced SpCas9 with *Francisella novicida* Cas9 (FnCas9, a Cas9 ortholog), which recognizes the same PAM sequence (NGG) but has different cleavage properties [75]. FnCas9 cleaves DNA 6–7 nucleotides upstream of the PAM sequence (instead of 3–4 bp for spCas9), increasing the distance between the cleaved DNA and the NGG PAM site. Although various Cas9 variants or orthologs have been utilized to expand the range of targetable sites for PE editing, current variants exhibit low editing efficiency and fidelity in general.

Recently, Liang et al. created a new PE system (CPE) using circular RNA and Cas12a, which is a subtype of Cas12 endonucleases. Compared to Cas9, Cas12a has a wider variety of sequence editing capabilities since it can recognize thymidine‐rich PAM sequences [76]. After screening and comparing Cas12a from other genera, they chose the Lachnospiraceae bacterium ND2006 Cas12a (LbCas12a) and its D156R‐H759A‐R1138A mutant as a nickase for constructing the CPE system. Four CPE systems using Cas12a (including niCPE:nickase‐dependent CPE, nuCPE:nuclease‐dependent CPE, sniCPE:split nickase‐dependent CPE, and snuCPE:split nuclease‐dependent CPE) were generated, and the editing efficiency at four different cellular loci was compared. The results revealed that SniCPE2 achieved the best editing level at three loci, with a maximum efficiency of 40% achieved. The editing efficiency of niCPE3/sniCPE3 did not significantly improve via adding ngRNA, and the niCPE and sniCPE systems can alter four genes simultaneously. Because of increased efficiency and PAM flexibility, this Cas12a‐based CPE system offers an innovative approach to extending the application of PE editing in the future [77]. However, it is noteworthy that the use of these Cas9 variants comes with several risks and challenges, including low cleavage activity, high off‐target propensity, and low affinity between Cas protein and reverse transcriptase. Although there are technologies, such as the high‐fidelity SpCas9 enzyme variant SpCas9‐HF1 [78], available to address these issues, it has not yet been proven that these technologies can be applied to PE systems.

#### 2.3.2 PE Variants for Large Insertions or Deletions

**FIGURE 2:**
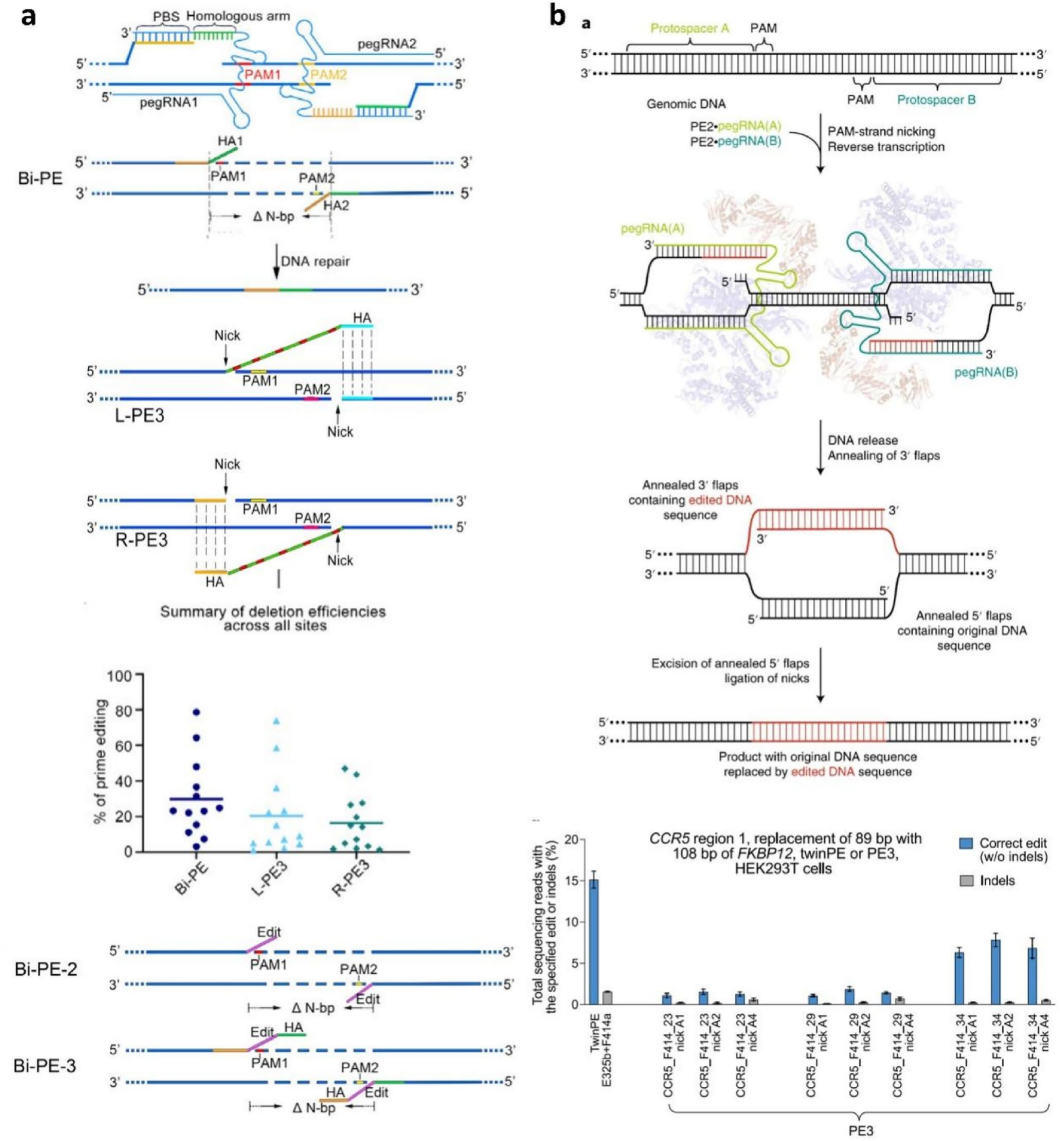
PE variants for large deletions and insertions. (a) Schematic illustration of Bi‐PE systems (top); the efficiencies of Bi‐PE‐mediated deletions at multiple endogenous locations in HEK293T cells (middle); schematic illustration of Bi‐PE‐2 and Bi‐PE‐3 methods (bottom). Copyright 2022, Oxford University Press. (b) Schematic illustration of Twin‐PE‐mediated gene editing (top); comparison of PE3 and Twin‐PE systems for insertion of FKBP coding sequence fragments at the HEK locus. Copyright 2022, Springer Nature.

The canonical prime editors have been shown to allow precise insertions of up to 40 bp and deletions of up to 80 bp in mammalian cells but are unable to mediate larger genetic alterations (e.g., > 100 bp). To extend the scope of large insertions or deletions, a new approach known as Bi‐PE was created by Tao et al. (Figure 2a). The Bi‐PE system consists of two pegRNAs, both of which have the intended edits and the homologous arms (HAs) in their 3′ flaps. They can hybridize and integrate into the genome, generating new DNA strands [79]. The Bi‐PE was found to be substantially more effective than the PE3 (L‐PE3: sgRNA upstream and R‐PE3: sgRNA downstream) for the deletion of large DNA fragments at various genomic loci (e.g., the *HEK3* loci) in HEK293T cells, with a low occurrence of unintended indels. Notably, the largest fragment deleted reached up to 1522 bp with an efficiency of 36.7%. Moreover, the researchers have also developed two additional variants, Bi‐PE‐2 and Bi‐PE‐3. The pegRNA of Bi‐PE‐3 includes the target editing sequences and HA regions with a length from 8 bp to 50 bp, which are homologous to the downstream DNA sequences. And the pegRNA of Bi‐PE‐2 includes only the editing sequences without any HA regions (Figure 2a). The activity of the Bi‐PE‐2 was found to be lower than that of the Bi‐PE‐3, suggesting that the presence of homologous arms can boost the editing efficiency of Bi‐PE systems.

Using a similar strategy, Liu and co‐workers reported the Twin‐PE system (Figure 2b), which uses a pair of pegRNAs encoding complementary 3′ flap templates that contain an overlap of over 20 nucleotides [39]. During the editing process, the 3′ fragments lacking any sequence similarity to the genome are generated and subsequently hybridize with each other. The unedited homologous double strand is then excised, facilitating the integration of a DNA strand into the genome. Due to this feature, the Twin‐PE is able to achieve higher efficiency for large insertions or deletions, endogenous sequence replacement, exonic mutation renewal, and large recombinase‐mediated inversions, with considerably fewer unintended byproducts compared to PE2 and PE3. Of particular note is its efficiency in targeted knock‐ins, where the Twin‐PE can insert a fragment up to 5.6 kb along with a site‐specific serine recombinase. In addition to Bi‐PE and Twin‐PE, other PE variants have also been reported to mediate efficient insertions or deletions of large fragments, including GRAND [80], PRIME‐Del [81], PEDAR [82], PASTE [83], and TJ‐PE [84]. Although most of these variants use paired pegRNAs to extend the editing scope, it is worth noting that paired pegRNAs still have limitations in terms of precision and off‐target effects.

A recent study reported the WT‐PE system that uses the wild‐type Cas9 nuclease instead of the Cas9 nickase, allowing for larger‐scale genomic modifications [35]. WT‐PE generates a double‐stranded break and a 3′ extended flap, which distinguishes it from previous PE systems. Additionally, the authors utilized a pair of pegRNAs with homologous arms together with WT‐PE to achieve a large‐scale targeted deletion (up to 16.8 megabase pairs) and internal molecular manipulations in various cell lines. These advances expand the versatility and application potential of PE editing.

Inspired by the efficient genomic insertion mechanism of retrotransposons, Zheng et al. developed a template‐jumping (TJ) PE approach using single pegRNA. Targeted initiation of reverse transcription (TPRT) is an important characteristic of non‐long terminal repeat retrotransposons that replicate and integrate into the genome. TJ‐pegRNA was engineered to mimic TPRT. The 3′ end of TJ‐pegRNA comprises RTT, PBS1, PBS2, and tevopreQl motifs. The cleaved DNA flap hybridizes with PBS1 to synthesize the first DNA strand utilizing the RTT. PBS2 hybridizes to a secondary cleavage site produced by sgRNA to commence template jumping and the production of the second DNA strand. TJ‐PE exhibits average efficiencies of 50.5%, 35.1%, and 11.4% for 200‐, 300‐, and 500‐bp insertions, respectively, which are 19‐ to 35‐fold superior than the efficiency of PE. It successfully inserted and expressed approximately 800 kb of the GFP gene in cells. The development of this technology has resulted in significant breakthroughs in gene editing, particularly in the insertion of large DNA segments and gene therapy [84].

To enhance the editing capacity of large DNA fragments by combining recombinases, Pandey et al. developed a technique called phage‐assisted continuous evolution enhances prime‐editing‐assisted site‐specific integrase gene editing (PASSIGE). It integrates the characteristics of PE with site‐specific large serine recombinases (LSRs), which can integrate DNA cargo exceeding 10 kilobases to accurately target particular sites in the mammalian genome. Multiple variations of Bxb1 were generated utilizing the phage‐assisted continuous evolution (PACE). The optimal variants were single mutants designated evoBxb1 and triple mutants eeBxb1, which exhibited enhanced target large DNA integration efficiencies (2.7‐fold and 4.2‐fold) compared to PASSIGE (WT Bxb1) across 12 mammalian genome loci, and surpassed another analogous method, PASTE, by around 9.1 to 16‐fold. This method dramatically enhances the efficiency of targeted integration of large DNA in mammalian cells [85].

#### 2.3.3 PE Variants for Chromosomal Translocations

Genome editing agents such as ZFNs, TALENs, and Cas9 nucleases have been frequently utilized to generate chromosomal rearrangements in living cells and organisms for disease modeling. Inspired by the successful development of PE variants for large deletions and insertions, Kweon et al. created the PE2 nuclease‐based translocation and inversion (PETI) method using paired pegRNAs for efficient and programmable chromosomal translocations [86]. The results showed that the PETI enabled DNA recombination in episomal fluorescent reporters and chromosomal translocation at an endogenous genomic locus. In addition, several cancer‐associated translocations and inversions (e.g., NPM1‐ALK and EML4‐ALK) were also generated by the PETI method in human cells, indicating the great potential of PETI in the development of disease models and novel therapeutics. In an independent study, Tao et al. created a similar system called bi‐WT‐PE, which also used paired pegRNAs with complementary sequences at the 3′ end and wild‐type Cas9 nuclease [35]. The bi‐WT‐PE was able to mediate large fragment deletions up to 16.8 megabase (Mb) pairs and targeted inter‐chromosomal translocations in HEK293T cells.

#### 2.3.4 PE Variants With Other Functions

The simultaneous editing of multiple genomic loci is highly demanded for the treatment of polygenic diseases. However, the multiplexing capability of current prime editors is limited and requires large expression constructs containing multiple gRNAs. For this purpose, Yuan et al. developed drive‐and‐process (DAP) arrays for multiplexed PE [87]. Tandemly constructed transfer RNA (tRNA)‐gRNA arrays are expressed in the DAP system, and the endogenous tRNA processing apparatus facilitates the release of the individual gRNAs. Specifically, a 75‐nt human cysteine tRNA (hCtRNA) has been engineered for the DAP arrays, enabling multiplexed PE of up to three loci with comparable or even higher efficacy than canonical PE. In addition, the DAP system was able to be packaged into a single lentivirus for precise 6‐bp deletion in the BCL11A gene, paving the way for future research on complex genomics and polygenic diseases.

Using a simple strategy, Jiao et al. developed the Random‐PE system that can introduce random sequences into the target sites of the genome in mammalian cells [88]. A pool of pegRNAs containing up to 10 bp random sequences was co‐delivered with prime editor and nick sgRNA, achieving an insertion efficiency of up to 39.07%. In addition, the Random‐PE was also utilized for in vivo gene evolution, where single or multiple mutations were introduced into the DNMTI, Actin‐b, and VEGFA genes. These results suggest that the Random‐PE system possesses great potential in diverse applications, such as in situ construction of PAM libraries, screening of enhancers for gene editing, and DNA barcoding.

## 3 Delivery of PE Systems

The successful delivery of PE components into target cells is crucial for achieving the desired gene modifications, making it a key factor in the treatment of genetic diseases [89]. The following sections summarize and discuss current delivery strategies for prime editors, including physical methods, viral vectors, and non‐viral vectors [90, 91].

### 3.1 Physical Methods

#### 3.1.1 Electroporation

**FIGURE 3:**
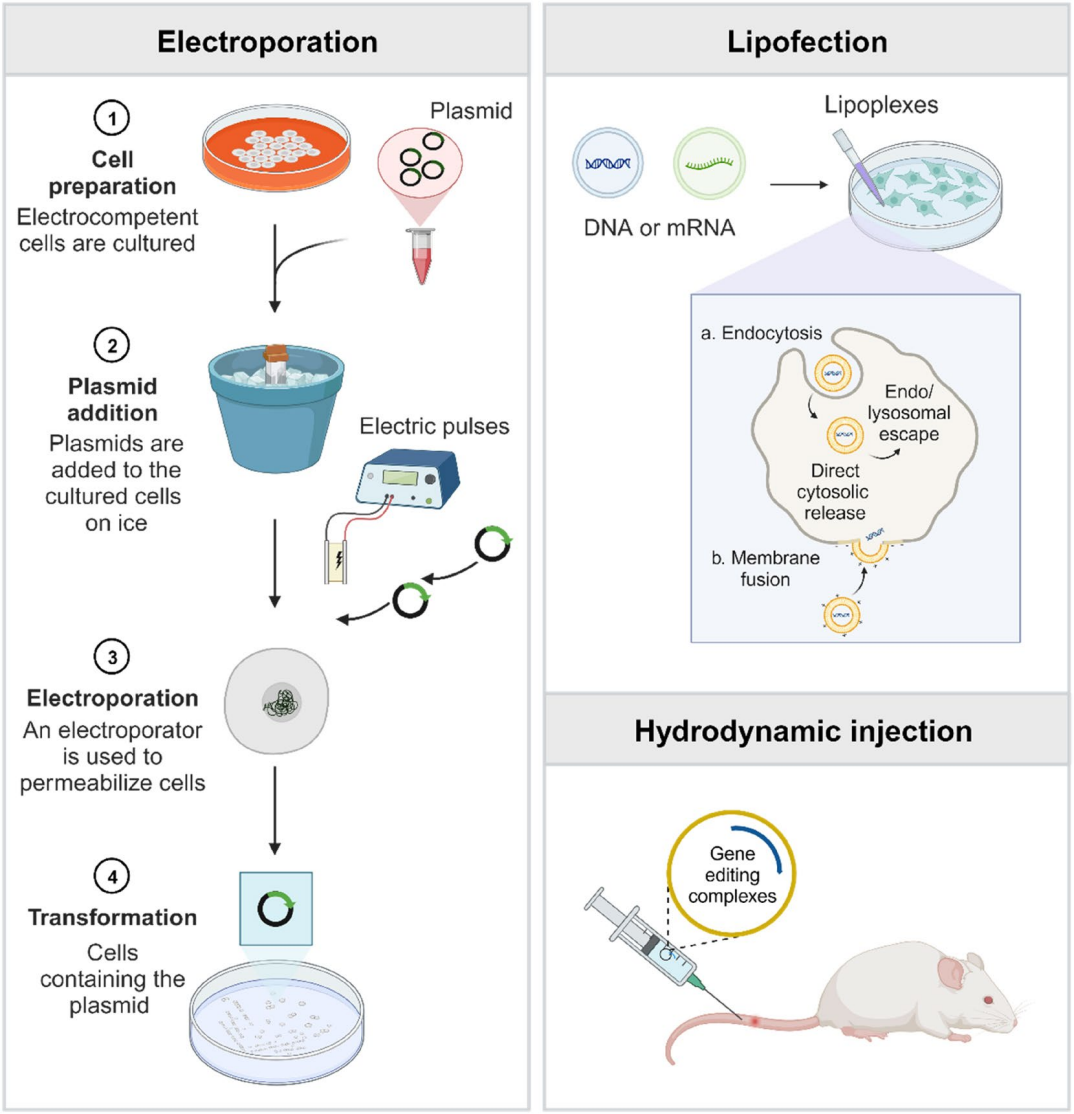
Physical methods are used to transfer the PE system into cells or mice. Electroporation is a technique that involves subjecting cells to short yet intense high‐field electrical pulses. The voltage difference created across the cell membrane during electroporation allows for the temporary formation of water channels or small pores in the membrane. These transient pathways enable the entry of exogenous macromolecules, such as DNA, RNA, proteins, and certain small molecules into the cell. Lipofection is a strategy that involves the use of cationic lipoplexes to encapsulate nucleic acids. Through endocytosis, these lipoplexes can be attracted to the negatively charged cell membrane and internalized by the cell, allowing exogenous nucleic acids to be delivered into cells. The hydrodynamic injection technique involves rapid injection of liquid drugs containing gene editing components into a blood vessel, typically in a living animal model. Created with BioRender.com.

**FIGURE 4:**
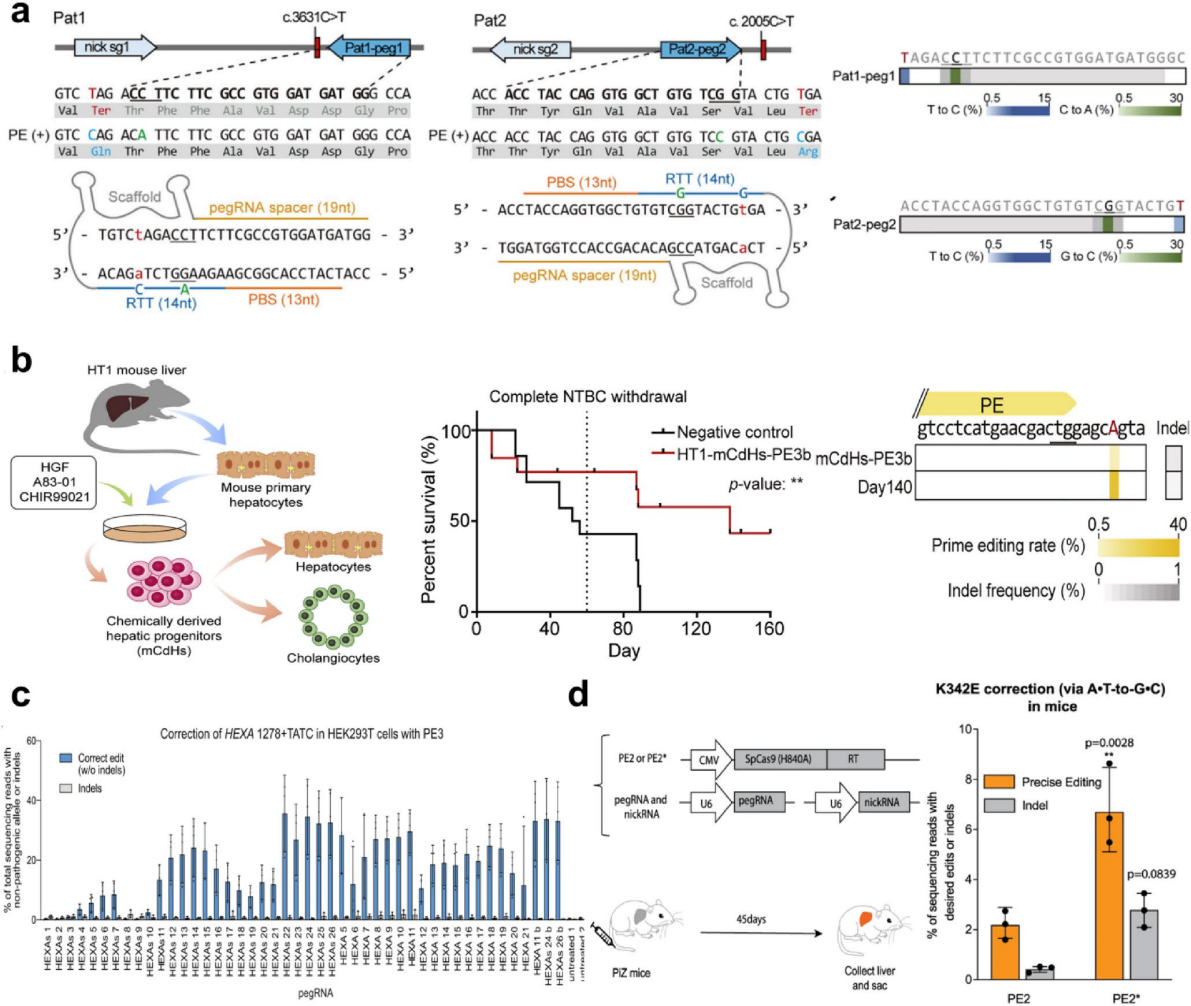
Disease‐causing mutations corrected by PE using physical methods. (a) The diagram shows the pegRNA‐ and gRNA‐targeting sites in the COL7A1 gene of the primary fibroblasts from two RDEB patients (left); the heatmaps demonstrate the editing rates of the *COL7A1* gene achieved by introducing plasmids expressing PE2, pegRNA, and gRNA by electroporation (right). Copyright 2022, Cell Press. (b) Schematic representation of the hepatocyte reprogramming technique utilized to produce chemically generated liver progenitor cells from HT1 model mice; the survival curves (middle) and the frequency (right) of the intended edits in the liver and HT1‐mCdHs‐PE3b cells 140 days after the transplantation of PE3‐treated cells. Copyright 2021, Cell Press. (c) HEK293T cells containing the *HEXA* ^*1278+TATC*^ allele mutation in Tay‐Sachs Tay disease were transfected with Lipofectamine 2000 encapsulating PE3 or PE3b system to generate a shifted 4‐bp deletion for correcting the pathogenic allele back to wild‐type. Copyright 2019, Springer Nature. (d) Schematic illustration of *AATD* gene correction mediated by hydrodynamic tail veil injection of PE systems in the PiZ transgenic mouse model with the *SERPINA1* E342K mutation (left); Comparison of the efficiency of K342E correction and indels in the liver of mice treated with PE2 or PE2* (right). Copyright 2021, Springer Nature.

Electroporation is a simple and efficient method for transferring gene editing tools into recipient cells through a transiently increased cell‐membrane permeability induced by an electric pulse (Figure 3) [92, 93]. Electroporation has been widely used to assess the editing capacity of PE systems in vitro [31, 34, 36]. For instance, Hong et al. used the electroporation method to treat recessive dystrophic epidermolysis bullosa (RDEB) in primary fibroblasts derived from two South Korean RDEB patients [34]. EB is a group of genetic disorders characterized by skin fragility and has four subtypes: RDEB, EB simplex, junctional EB, and kindler EB. Among them, RDEB, an autosomal recessive dystrophic EB, is the most severe subtype raised from mutations in the *COL7A1* gene encoding type VII collagen. The aberrant changes destroy the anchoring fibrils and further disrupt the functions of the normal skin, leading to blistering and ulceration even from minor skin injuries [94, 95]. To correct the mutated gene, two pegRNAs were designed, and the editing capacity was reached up to 10.5% with PE3/Pat1‐peg1 and 5.2% with PE3/Pat2‐peg2, with a low undesired editing rate (~1%). And functional type VII collagen was restored in the fibroblasts of patients (Figure 4a) [34]. Kim et al. also employed the PE system to treat hereditary tyrosinemia type 1 (HT1) by electroporation [31]. HT1 is caused by a G‐to‐A mutation in the *Fah* exon 8, which induces a deficiency of the fumarylacetoacetase (FAH) enzyme and severe liver damage [96]. The researchers tested the efficiency of PE3 and PE3b in mediating the A‐to‐G conversion in CdHs chemically reprogrammed from the primary hepatocytes of HT1 model mice. The results showed a sufficient average efficiency of 2.3% without any bystander effects. Moreover, transplantation of the edited HT1‐CdHs population into HT1 mice resulted in significantly longer survival profiles compared to the control group (Figure 4b) [31]. It should be noted that electroporation can cause some side effects on the treated cells, such as potential mechanical damage and noticeable changes in cell phenotype, which restrict its clinical applications. Although electroporation can also be employed in vivo by directly applying the electrode surface to the target tissue, conducting in vivo clinical electroporation studies in humans presents challenges due to the difficulty of precisely adjusting electroporation parameters and predicting potential tissue damage [91].

Cystic fibrosis (CF) is an autosomal recessive condition resulting from a three‐base‐pair deletion (F508del) in the *CFTR* gene. Sousa et al. used multiple innovative enhanced PE approaches to promote *CFTR* F508del correction, incorporating epegRNA, PEmax, MLH1dn protein co‐expression, silent edits, PE6, and dsgRNA to modulate the chromatin configuration of the target locus. The optimized measures significantly enhanced editing efficiency, attaining 58% correction efficiency in 16HBEge‐F508del cells and 25% correction efficiency in primary airway epithelial cells from cystic fibrosis patients via electroporation. Furthermore, minimal non‐target editing was observed following the use of the optimized PE method, indicating that PE exhibits a high accuracy [97].

#### 3.1.2 Lipofection

Lipofection is a commonly used method for delivering gene editing tools and other genetic materials into cells by cationic lipid‐based transfection reagents (Figure 3). The process of lipofection begins with the preparation of complexes consisting of gene editing components and cationic lipids. Positively charged cationic lipids interact with the negatively charged genetic materials to form lipoplexes. The lipoplexes protect the genetic cargoes from degradation and facilitate their entry into the target cells. Commercial lipofection reagents such as Lipofectamine 2000 and Lipofectamine Messenger MAX are commonly used for intracellular delivery of PE systems (either plasmid or mRNA formats) in vitro [29, 56, 98]. Anzalone et al. tested the editing efficiency of PE3 using Lipofectamine 2000, where > 50% gene modification was achieved in multiple cell lines. Regarding the Tay‐Sachs disease caused by a 4‐bp insertion in the *HEXA* gene, Anzalone et al. screened 43 pegRNAs and 3 ngRNAs in HEK293T cells carrying the *HEXA* ^*1278+TATC*^ mutation using PE3 lipofection and achieved a median editing efficiency of over 20% (maximum 33%) with a low indel rate of only 0.32% (Figure 4c) [23]. Nelson et al. improved the effectiveness of canonical pegRNAs and generated a novel engineered pegRNA (epegRNA) with the structural motif in an independent study [53]. epegRNAs were also employed in plasmid lipofection and nucleofection to treat Tay‐Sachs disease in *HEXA* ^*1278+TATC*^ HEK293T cell models and primary patient‐derived fibroblasts.

#### 3.1.3 Hydrodynamic Injection

Hydrodynamic injection is a powerful method for directly delivering gene editing tools and genetic materials into target tissues or organs via intravenous (i.v.) administration (Figure 3) [99]. By controlling the pressure in capillaries, the hydrodynamic method can enhance the permeability of parenchymal cells through rapid i.v. administration of a large volume of fluid. This process disrupts endothelial barriers, allowing exogenous molecules to enter cells, where they become trapped over time [100, 101]. Using this method, the plasmid DNA can be delivered successfully to the mouse liver [101, 102]. Jang et al. directly delivered plasmids encoding the PE3 system into HT1 mice by hydrodynamic injection [33]. The average frequency of FAH^+^ cells in the liver and desired edits can reach 61% and 11.5% at day 40 due to selective amplification of edited cells, respectively. The indel level of PE at the target site was only 0.78% on average, far below Cas9‐directed HDR (26%) and ABE (1.9%), demonstrating the high accuracy of the PE strategy. Similarly, hydrodynamic injection has been employed in the treatment of another genetic disease of the liver. A pathogenic E342K mutation in the *SERPINA1* gene, which induces lung and liver diseases, is the cause of alpha‐1 antitrypsin deficiency (AATD). Patients with a pure mutation have aggregates of PiZ proteins in hepatocytes and a lack of functional AAT proteins in the lungs. Liu et al. used hydrodynamic injection to deliver PE2 or PE2*, pegRNA, and sgRNA plasmids to mouse models of PiZ [30]. Following 45 days, deep sequencing results revealed that the optimized PE2* system had a greater average gene repair capacity (6.7%) than PE2 (2.1%) with a reduced indel frequency (0.4%–2.7%) (Figure 4d). Although hydrodynamic injection has shown promise in preclinical studies, there are several disadvantages and challenges that hinder its clinical translation, including potential toxicity, inconsistent transfection efficiency, and limited tissue targeting [103, 104, 105, 106, 107].

### 3.2 Viral Delivery Vectors

**FIGURE 5:**
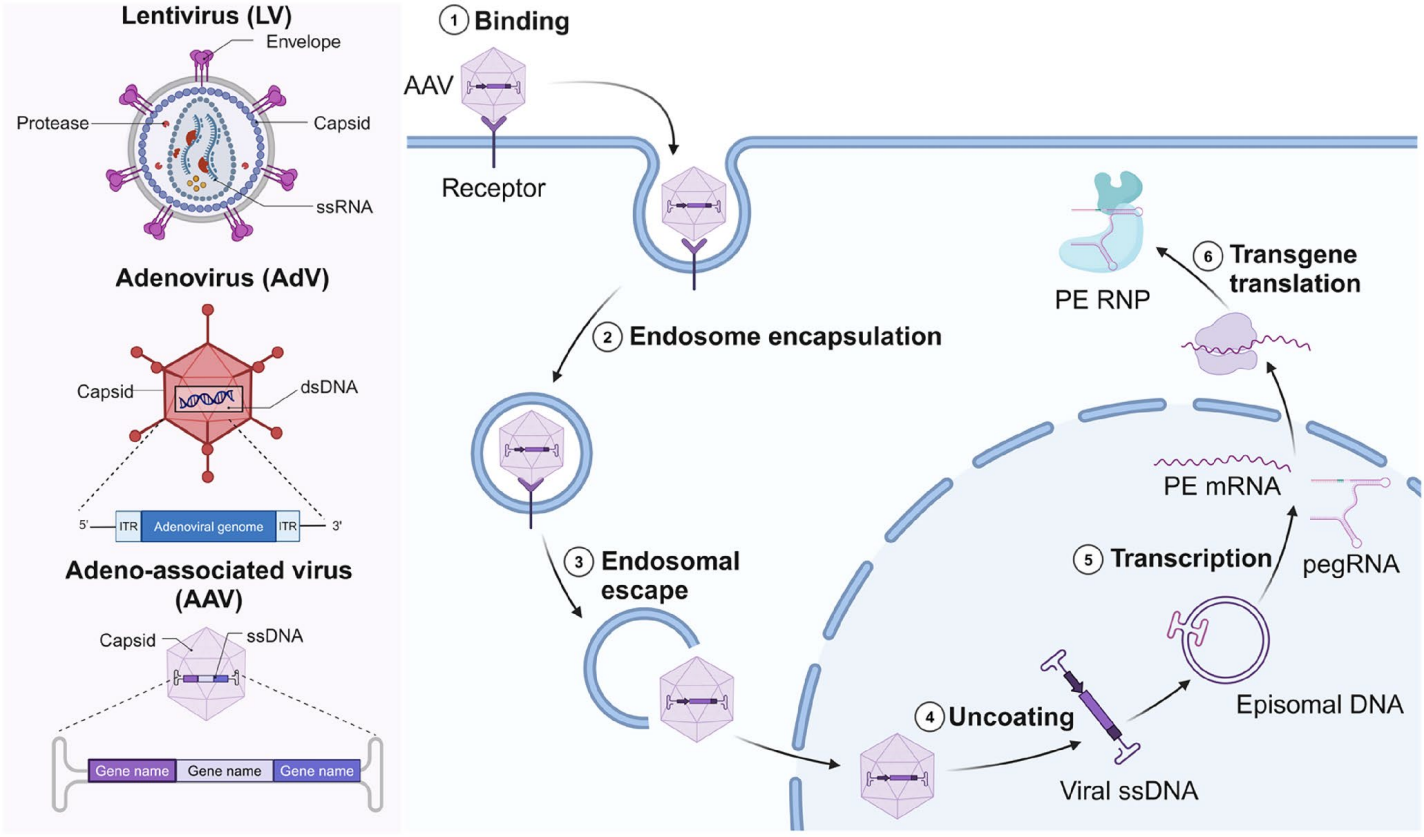
Viral delivery vectors for the delivery of PE systems. Three types of commonly used viral vectors include lentivirus (LV), adenovirus (AdV), and adeno‐associated virus (AAV) (left); schematic illustration of the cellular infection process mediated by AAV (right). Created with BioRender.com.

Viruses have naturally evolved efficient mechanisms for replicating themselves and assembling offspring within a short time after invading their hosts. These mechanisms have been used to engineer viruses in order to construct artificial viral vectors for the delivery of therapeutic molecules. Viral vectors are one of the most widely used vectors in gene therapy research due to their exceptional efficacy [108]. Numerous viral vectors have been developed for in vivo gene therapy applications and are progressively moving into clinical development (Figure 5).

#### 3.2.1 Lentiviral Vector

Lentiviral vectors (LVs), derived from the human immunodeficiency virus (HIV), have the ability to integrate into non‐dividing cells and can reversely transcribe exogenous RNA, resulting in the random integration of an exogenous gene into the genome of targeted cells [109, 110]. LVs have a large cargo packaging capacity (~10 kb) achieved by deleting a large proportion of the original virus sequence and splitting the intended residue into multiple constructs [111]. However, the tropism of LVs is limited, although several pseudotypes are also available by enveloping special proteins that derive from other viruses [112, 113]. Jang et al. constructed a lentiviral library containing hundreds of pegRNAs for screening in HEK293T cells [33]. Another notable disadvantage is that LV‐delivered genes can exhibit long‐term expression after delivery, leading to detrimental off‐target byproducts [114]. Furthermore, lentiviral vectors serve as integrating vectors that allow for the long‐term expression of transgenes. This integration process has the potential to create chimera genes, which are composed of both lentiviral proviral and host genome sequences. As a consequence, abnormal splicing of cellular transcripts may occur, making LVs less commonly used as carriers for in vivo gene editing studies.

#### 3.2.2 Adenoviral Vectors

**FIGURE 6:**
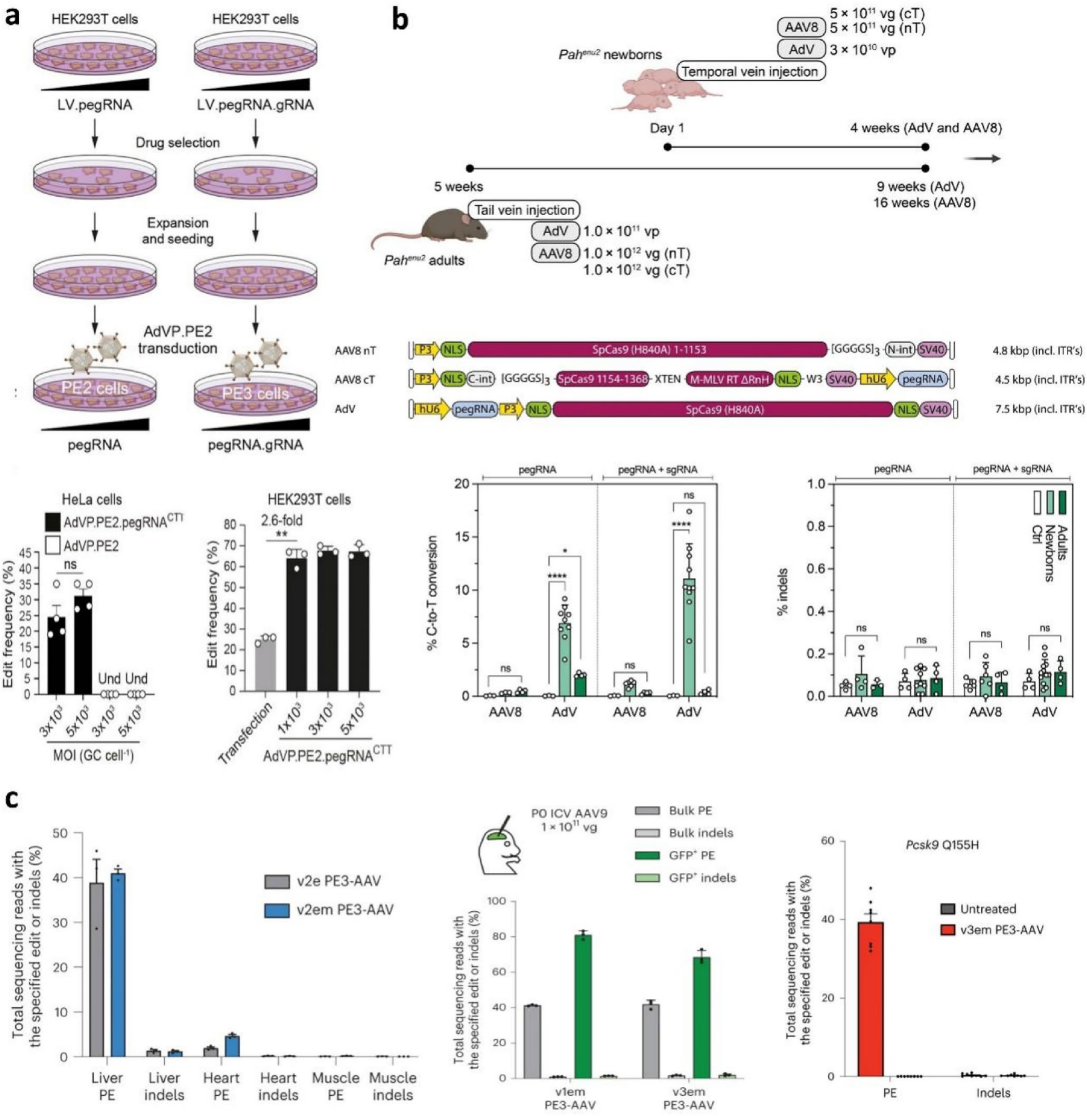
Representative strategies to correct disease‐causing mutations by viral delivery vectors. (a) Schematic illustration of the generation of HEK293T cells stably expressing pegRNAs or pegRNA/gRNA pairs by LV and AdV transduction of the resulting cell populations (top); the frequency of editing by co‐transfecting plasmids encoding PE2 and pegRNA into HeLa cells and HEK293T cells with different multiplicity of infection (bottom). Copyright 2021, Oxford University Press. (b) Schematic outline of the experimental setup for correction of pathogenic *Pah* ^*enu2*^ mutation in newborn and adult PKU mice by delivering AAV vectors or AdV vectors encoding intein‐split PE2 or unsplit PE and pegRNA (top); the genomic mapping reveals the presence of AAV vectors with intein‐split PE2^ΔRnH^ and mPKU pegRNA (middle); the rates of intended correction and indels of the *Pah* ^*enu2*^ allele in newborn and adult PKU mice after AAV or AdV vector‐mediated delivery (bottom). Copyright 2022, American Association for the Advancement of Science. (c) The HTS sequencing of PE in the liver, heart (left), and brain (middle) at the *Dnmt1* + 1 C‐to‐G via spilt AAV vectors; the edit efficacy of disease‐associated loci utilizing an optimized dual AAV systems (right). Copyright 2023, Springer Nature.

Adenoviral vectors (AdVs), belonging to the adenoviridae family and associated with various human diseases, have been extensively studied for over 50 years [89, 115, 116, 117]. Different serotypes of AdVs have been employed for PE in human cells [118, 119, 120]. For example, Wang et al. constructed a fully viral gene‐deleted adenoviral vector containing exclusively recombinase to deliver the plasmid of the PE2 system in HeLa and HEK293T cells, yielding high PE frequencies [96]. The results demonstrated the potential of AdVs for the all‐in‐one delivery of full‐length functional PE complexes into cells and model animals (Figure 6a). Böck et al. established an AdV‐mediated PE approach to correct phenylketonuria (PKU) [29], which is caused by a mutation (C‐to‐T conversion) in the *Pah* gene and induces the deficiency of phenylalanine hydroxylase and the accumulation of excessive phenylalanine in the blood [29, 121]. They evaluated the correction efficiency of PE in HEK293T cells and identified the best pegRNA (19.7% with PE2) for the subsequent in vivo experiments. They then delivered the PE3 system along with an additional sgRNA to the model mice using AdVs and achieved the highest correct rate of 22.2% in vitro and 14.4% in vivo without detectable off‐target mutations. This led to a significant reduction in blood phenylalanine levels in the treated mice (Figure 6b). However, the successful infection of AdV relies on the presence of integrins and CAR proteins in the target tissues and cells. Simultaneously, in vivo administration of AdVs can trigger the innate immune response as well as the production of inflammatory cytokines and viral‐derived proteins, limiting the efficacy and durability of gene editing therapies. To prevent immunological reactions to AdVs, scientists currently employ many strategies, including the utilization of uncommon human serotypes that have low frequencies of seropositivity as well as a range of non‐human AdV vectors to minimize the occurrence of cross‐reactive immunity [122].

#### 3.2.3 Adeno‐Associated Viral Vectors

Nowadays, adeno‐associated viral vectors (AAVs) have become widely used as gene delivery vehicles in lab and clinic trials because of their high biocompatibility and efficient tissue targeting capabilities [123]. The AAV, originating from the parvovirus family, relies on co‐infection with other viruses for replication due to its own replication deficiency [124]. It contains ~4.7 kb of single‐stranded DNA consisting of three important genes (Rep, Cap, and Aap), three open‐reading frames, and two inverted terminal repeats. Similar to AdV, AAV has different serotypes and can target specific tissues and organs [125]. However, the significant defect of AAV is its restricted packaging capacity, which limits the size of the gene payload it can carry. AAV can only accommodate cargo of approximately up to 5 kb, whereas the size of the prime editor along with the pegRNA exceeds its packaging capacity [124, 125]. To address this issue, Liu et al. designed a split PE system (sPE) to facilitate the delivery of prime editors by separating the nCas9 from the RT domain. Subsequently, the system is combined with a dual adeno‐associated virus (AAV) system, wherein the first AAV vector is responsible for expressing the nCas9, while the second AAV vector expresses the RT, pegRNA, and nicking sgRNA. The study demonstrated that the implementation of this optimized system effectively reduced the illness phenotype shown in the mouse model of tyrosinemia type I. Besides, researchers have developed a novel dual‐AAV strategy to overcome the size limitation, wherein the gene editing agents are split into two AAV vectors and co‐transduced into target cells [126]. These two AAV vectors carried a separate part of the editing tool and reconstituted full‐length gene editing agents through genome recombination and mRNA or protein trans‐splicing strategies, dramatically increasing the capacity of AAV vectors up to 14 kb. This split‐intein dual‐AAV method has been successfully tested in mouse models with various diseases [127, 128, 129]. Böck et al. achieved the desired G‐to‐C conversion in the *Dnmt1* locus with editing rates of 14.4% using intein‐split PE2‐AAV, albeit lower than AdV‐mediated editing with unsplit PE2 on a single vector (Figure 6b) [29]. Liu et al. also adapted two AAV8 particles, each containing N‐ or C‐terminal truncated PE2 segments, for correcting the alpha‐1 antitrypsin deficiency, resulting in a precise efficacy of 3.1% at 10 weeks [30].

The lower editing efficiency of split AAV vectors may be attributed to the challenging design of dual‐AAV and the uncontrollable intermolecular interaction required for recombining full‐length prime editors. Increasing the co‐expression of proteins in cells may enhance editing efficiency [53]. Recently, Davis et al. developed an optimized dual AAV system, v1em and v3em PE‐AAV [130]. They snipped at the PE gene's ideal location and divided the PE gene into two pieces that were then separately encapsulated into two AAV vectors. They found that mRNA transcript stabilization cis‐elements on the AAV genome are necessary for effective PE editing. In addition, they further optimized the AAV genome structure by changing the promoter and inserting benign or quiet mutations near the target editing site to bypass intracellular MMR. In terms of the PE system, they improved the codon for M‐MLV RT and adjusted the 3′ end of pegRNA to increase stability. These manipulations led to effective editing in a number of mouse organs in vivo, including the brain (42%), liver (46%), and heart (11%). This editing ratio was effective enough to reach therapeutically relevant levels without significantly increasing off‐target events or liver injury markers (Figure 6c). This approach further strengthens the potential of AAV vectors for the delivery of gene editing agents in basic research and therapeutic applications. LCA is a rare monogenic genetic disease affecting visual function. Mutations in the retinal pigment epithelium‐specific protein 65 kDa (*RPE65*) gene account for a significant percentage of LCA cases (5%–10%) [131, 132]. By sub‐retinal injection of AAV‐PE2 into rd12 mice that serve as a disease model for LCA, an average efficiency of 6.4% was achieved without unintended byproducts, leading to the restoration of visual functions in the mouse models [33]. Nevertheless, in vivo PE using AAV still faces several limitations that need to be addressed, such as limited packaging capacity, low editing efficacy, and immunogenicity [133, 134, 135]. AAV is unable to replicate continuously, and the majority of virus DNA remains as stable fragments within cells without integrating into the host genome. This poses a low risk of insertion; however, insertion mutations can still occur at higher doses. Recent evidence has demonstrated that AAV vector genomes, containing CRISPR components, can integrate into the genome of the host cell at locations where double‐strand breaks occur [136]. This finding has sparked public apprehension regarding the efficacy and long‐term safety of AAVs. In addition, the packaging efficiency of AAVs greatly reduces when the length of the cargo exceeds 5 kb, and the limited capacity for packaging restricts the broad use of AAVs. Moreover, humans naturally harbor wild‐type AAVs and often contain antibodies and memory T cells specific to the AAV capsids. This can result in the neutralization of antibodies and the activation of cellular immune responses, which can decrease the effectiveness of the treatment [134]. Following the administration of AAV vectors, the cellular responses of the immune system are activated, which results in the infiltration and destruction of the transfected cells, ultimately reducing the effectiveness of the therapy. Nevertheless, the importance of tailored delivery of AAV vectors in vivo should not be underestimated. While AAV vectors have the ability to infect several cell types [137], not all serotypes of AAVs can efficiently target specific tissues or organs. Therefore, additional research is required to investigate the targeting capabilities of AAVs. Despite the promising application prospects of dual‐AAV strategies in gene editing and therapy, several limitations exist. The strategy relies on the recombination of two AAV vectors within cells to form a complete gene‐editing tool [138]. However, this recombination efficiency is often low. Additionally, dual‐AAV strategies have inherent disadvantages in production compared to single‐AAV strategies [139]. The production process requires higher doses, and the high content of impurities in the production process makes it difficult to meet market demands. Furthermore, dual‐AAV strategies may produce foreign proteins that trigger immune responses, such as bacterial proteins produced when using split intein. These issues may limit the safety and efficacy of dual‐AAV strategies in clinical applications. Ongoing research is focused on optimizing AAV delivery strategies and developing smaller PE systems to overcome these limitations and unlock the full potential of in vivo PE.

### 3.3 Non‐Viral Delivery Vectors

Non‐viral delivery offers an alternative option for delivering the PE system, leveraging its numerous advantages. Firstly, non‐viral vectors have a larger packaging capacity, allowing them to accommodate larger PE components for all‐in‐one delivery. Secondly, non‐viral vectors can be designed to minimize undesirable immune responses, improving the safety of PE‐based therapies. In addition, non‐viral vectors provide greater flexibility in their design and formulation and enable targeted delivery of PE components to specific tissues or cell types, increasing the precision and specificity of PE editing. Moreover, non‐viral vectors are often more amenable for large‐scale production and manufacturing for clinical applications. Therefore, using non‐viral vectors for the delivery of PE systems presents a promising approach for the development of effective and widely applicable PE therapies.

**FIGURE 7:**
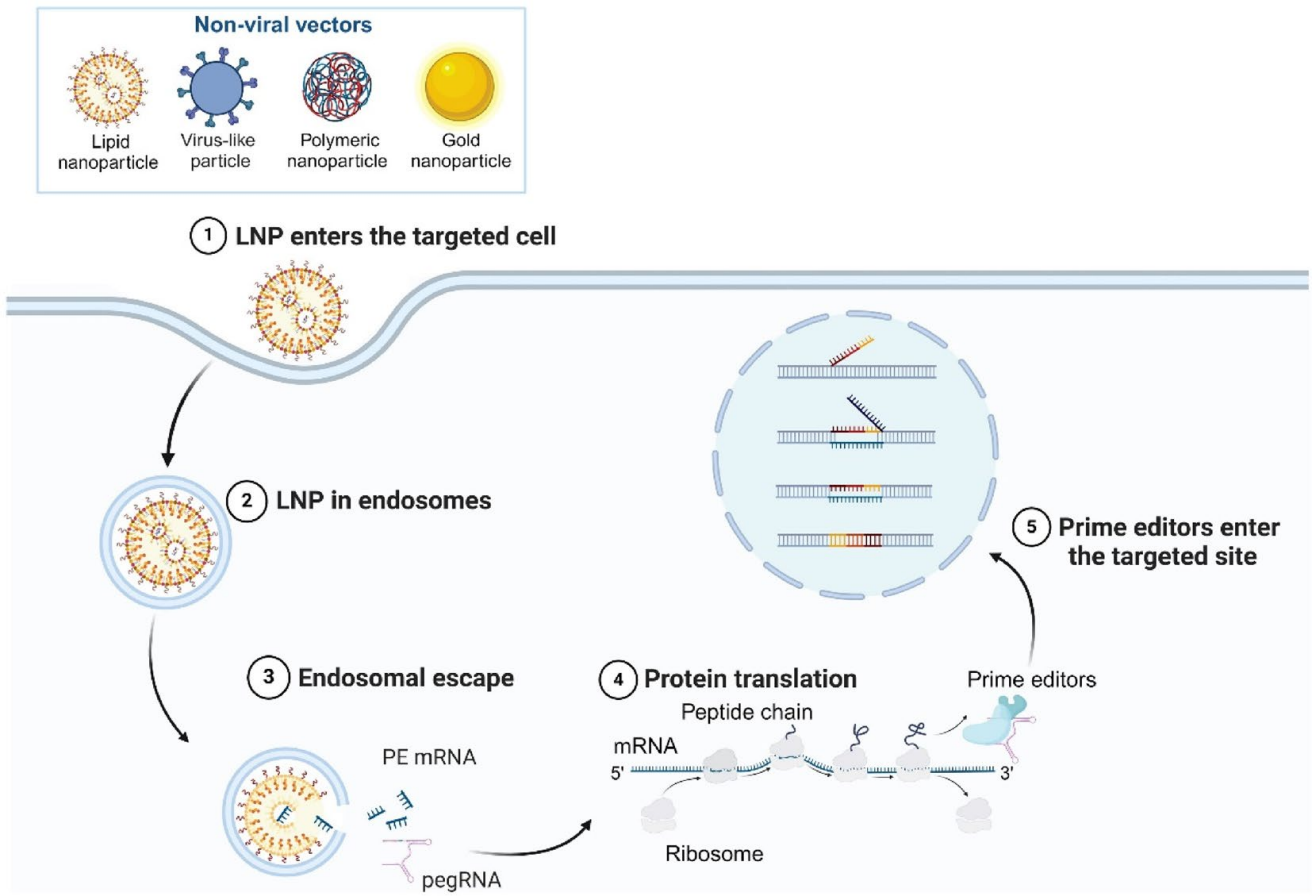
Representative non‐viral vectors and schematic illustration of intracellular delivery of prime editors using lipid nanoparticles as a sample. Created with BioRender.com.

At present, numerous non‐viral carriers, such as lipid nanoparticles (LNPs) [140, 141, 142, 143, 144], virus‐like nanoparticles (VLPs) [145], polymeric nanoparticles [146, 147], peptide‐based nanoparticles [148, 149], and inorganic nanoparticles [150, 151], have been explored for delivering gene editing agents such as CRISPR/Cas9 and base editors (Figure 7). Despite the successes achieved by the above gene delivery carriers, the development of efficient non‐viral delivery systems specifically tailored for prime editors remains a crucial step towards advancing PE as a therapeutic tool for genetic disorders and other biomedical applications. Like other genome editing agents, prime editors can be delivered in three formats: plasmid DNA (pDNA), mRNA, and prime editor/pegRNA ribonucleoprotein (RNP) [152]. Due to the relatively large size of the prime editor (PE) complex, direct delivery of RNP can be more challenging. Currently, the preferred strategies for delivering PE systems involve using non‐viral vectors to deliver pDNA or mRNA. In general, mRNA delivery offers several advantages over DNA‐based delivery, including faster action, transient expression, reduced off‐target effects, and a lower risk of insertional mutagenesis [152, 153].

**FIGURE 8:**
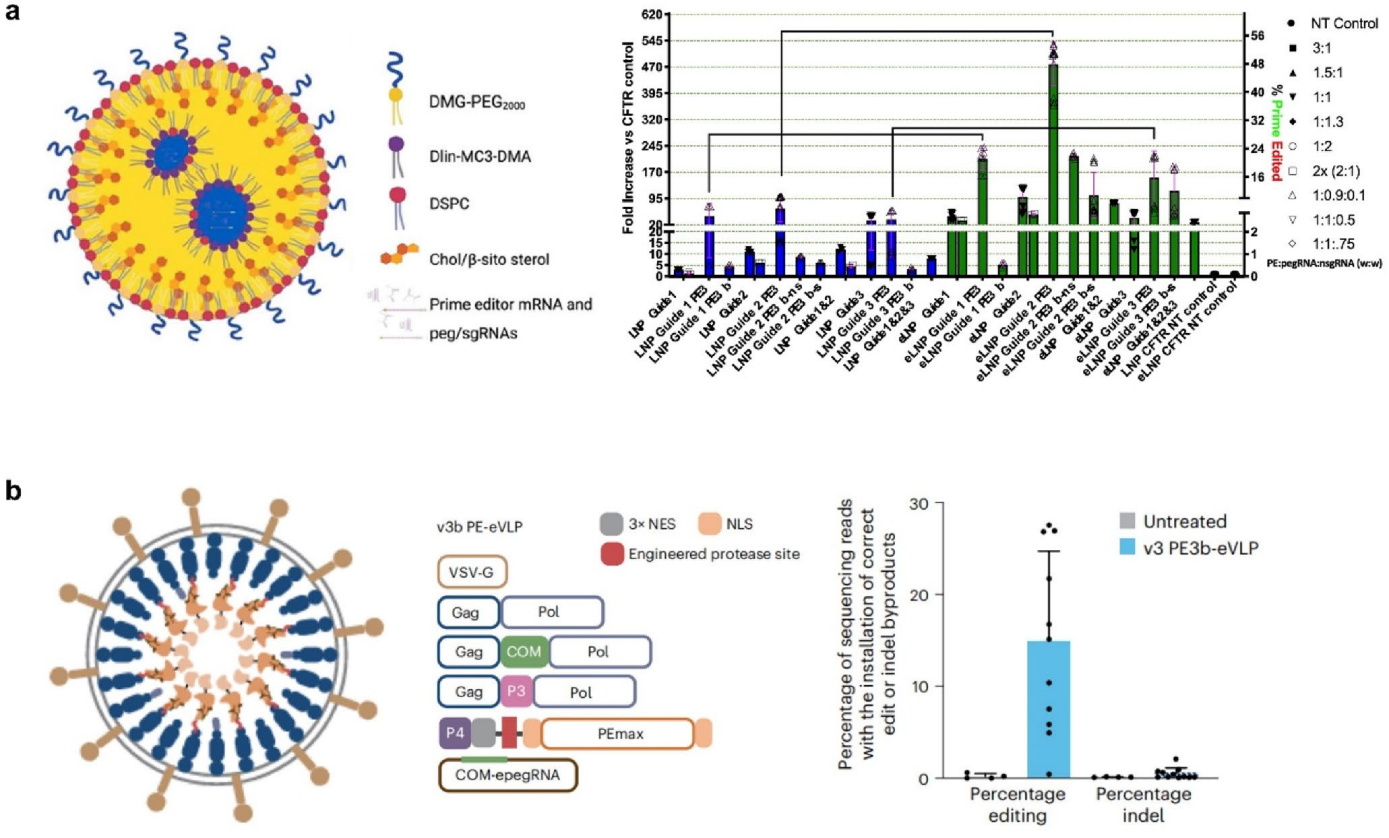
Representative strategies to deliver PE systems for correcting disease‐causing mutations. (a) Schematic diagram of each component of eLNP and the PE rates. Copyright 2023, Springer. (b) Representative PE‐eVLP architecture (left) and a PE editing of a 4‐bp deletion at the *Mfrp* gene in rd6 mice (middle); the retinal editing efficiency and the indel frequency of PE‐eVLPs (right). Copyright 2023, Springer Nature.

Lipid nanoparticles (LNPs), as one of the most efficient and clinically advanced non‐viral vectors, have been extensively used for delivering mRNA that encodes gene editing proteins in vitro and in vivo [154]. LNPs are typically composed of four lipid components, which are ionizable lipids, cholesterol, phospholipids, and PEGylated‐lipids [155, 156]. These components self‐assemble with mRNA to form nanoparticles, which are crucial for encapsulating mRNA cargoes and mediating efficient mRNA expression. LNPs have made significant progress in cell and animal models with genomic abnormalities by delivering gene editing tools for various applications, including CRISPR/Cas9 for gene knockout, and CRISPR/Cas/ssDNA as well as base editors for precise gene correction [141, 143, 144, 157, 158]. For instance, LNPs have been used to treat hepatic‐related disorders by delivering RNA based CRISPR/Cas system, such as transthyretin amyloidosis (ATTR), hereditary tyrosinemia, hypercholesterolemia, and human lipoprotein metabolism disorder [142]. Additionally, by altering administration routes, LNPs enabled repeated delivery of Cas9 mRNA and sgRNA into skeletal muscles, as demonstrated in a DMD mouse model [159]. Furthermore, LNPs can be engineered to deliver CRISPR/Cas systems to tumors for disrupting oncogenes. A recent study has shown that intraperitoneal injections of anti‐human epidermal growth factor receptor (EGFR) antibody‐modified LNPs achieved up to 80% gene editing in ovarian tumor mouse models, leading to suppressed tumor growth and extended survival rates [160]. Compared to CRISPR/Cas or base editor delivery, LNP‐mediated prime editor delivery is still in a relatively early stage. Herrera‐Barrera et al. first developed enhanced LNPs (eLNPs) and lipid‐like nanoparticle (LLN) to deliver PE3 mRNA, pegRNA, and nicking sgRNA (Figure 8a) [161]. The eLNPs, which incorporate β‐sitosterol to enhance the endosome escape efficiency of RNA cargoes, showed a remarkable PE efficiency of 54% in a reporter cell model. Most recently, Prime Medicine Inc. reported at the ESGCT (European Society of Gene and Cell Therapy) meeting that their targeted LNPs enabled precise editing of p.L348 in non‐human primates (NHPs) with efficiencies of up to 50% in the whole liver and up to 83% in the hepatocytes at day 14 without significant unintended edits and safety concerns following i.v. administration [162]. Instead of delivering PE mRNA, Chen et al. recently reported a novel LNP‐based platform that contains pH‐sensitive PEGylated lipids enabled the delivery of prime editor (PE2)/pegRNA ribonucleoproteins (RNPs) and achieved 1% PE editing efficiency in HEK293T cells [163].

Gene editing medications can also be delivered effectively with VLPs. The envelope and capsid, two typical viral proteins, are present in VLPs, but the viral genome is absent. VLPs can effectively and temporarily transfer mRNA or proteins to target cells for medication administration by leveraging the cell tropism of viruses. Up to now, VLPs have been employed in the manufacturing of viral vaccines. Recently, Meirui An et al. engineered VLPs to deliver the PE system in vitro and in vivo. Several modifications were made, which include (i) PE editor and pegRNA optimization; (ii) nuclear export signal insertion into gag‐PE to stop partial PE cleavage with gag; (iii) introduction of the MCP‐MS2 system to ease the pegRNA packaging by allowing epegRNA and ngRNA to be present in the same particle; and (iv) separation of gag from the PE editor and addition of two α‐helical peptides. Following the aforementioned adjustments, the in vivo editing efficiency was evaluated in a mouse model of ocular illness, and the best PE‐eVLPs achieved a 15% repair efficiency (Figure 8b) [164]. These results provide an important “proof‐of‐concept” for non‐viral‐mediated PE in vivo, paving the way for further development of PE‐based gene therapies.

Although non‐viral vectors provide significant advantages in delivering gene editing tools, they encounter several challenges. First, the in vivo stability and cycle duration of non‐viral vectors are rather brief, hence impacting the sustained expression of gene editing tools in vivo. In order to tackle this problem, scientists have developed strategies such as chemical modification to improve the durability of vectors [165], or creating vectors that can react in particular physiological or pathological circumstances, for example, being sensitive to changes in pH [166]. Secondly, the manufacture of non‐viral vectors on a large scale can face challenges related to cost and transportation stability. The therapeutic application of non‐viral vectors will be significantly influenced by the stability of these vectors during storage and transportation. Currently, gene therapy modalities encapsulated in non‐viral vectors are typically stored and transported frozen or refrigerated. However, the process of thawing or rewarming might potentially alter the physical and chemical characteristics of the vectors, leading to a decrease in their effectiveness [167]. In order to tackle this problem, scientists have been investigating lyophilized formulations to improve the durability of vectors [168].

## 4 Comparison of PE With Other Genome Editing Technologies

PE represents a significant advancement in genome editing technology, offering enhanced precision, versatility, and reduced off‐target effects compared to other genome editing technologies including CRISPR/Cas‐mediated genome editing and base editing.

### 4.1 Precision

PE is distinguished by its ability to delete, insert or modify genetic information at specific genomic sites without the need for double‐strand breaks or donor DNA templates. This capability provides greater precision than CRISPR/Cas genome editing, which depends on the cell's own DNA repair mechanisms and often results in unpredictable outcomes [169]. While base editing is highly precise in converting one DNA base to another, it is limited to specific types of base changes and lacks the ability to introduce insertions or deletions, a flexibility that PE offers.

### 4.2 Efficiency

Although the overall editing efficiency of PE is relatively low and variable depending on the genomic context and target sites, it offers a more controlled editing process crucial for therapeutic applications. In contrast, CRISPR/Cas systems, while generally more efficient, do not provide the same level of control [170]. In terms of base editing, while efficient within their specific scopes, are limited by the types of edits they can perform.

### 4.3 Off‐Target Effects

A significant advantage of PE is its reduced off‐target effects. Unlike CRISPR/Cas systems, which induce double‐strand breaks potentially leading to unintended indels, PE's mechanism inherently minimizes these risks. Compared to base editing, while base editing generally exhibits fewer off‐target effects related to DNA breaks, it can result in bystander editing—unintended modifications at adjacent bases that fit the editing criteria, representing another form of off‐target editing [22]. PE allows for more complex genetic modifications without substantially increasing off‐target activities or causing bystander effects, thereby enhancing its precision and safety.

### 4.4 Ease of Use

Currently, PE poses certain challenges in terms of delivery and protocol complexity, primarily due to the larger size of its editing components. This is in contrast to the more established and streamlined protocols available for CRISPR/Cas and base editing technologies. Improvements in delivery methods and vector design are critical for enhancing the practicality and applicability of PE, especially in clinical settings [24].

In summary, PE holds great potential for expanding the capabilities of genome editing, particularly in applications requiring high precision and minimal off‐target effects. As this technology continues to evolve, it is expected to play an increasingly important role in both research and therapeutic contexts, potentially surpassing existing genome editing methods.

## 5 Current Challenges of PE in Gene Therapy

### 5.1 Efficiency, Fidelity, and Off‐Target Effect of PE Systems

The efficiency and fidelity of PE systems remain major concerns. Currently, most validations of therapeutic applications of PE are performed at the cellular level, with low editing efficiency in general. Moreover, the editing efficiency of PE systems varies significantly between different target sites and cell types [23]. Therefore, improving the overall editing efficiency of PE is essential to satisfying therapeutic outcomes. Understanding the key factors that affect editing efficiency will be important in expanding the scope of PE editing. Several attempts have been made to modify and optimize the basic components of PE or incorporate foreign molecules (such as nicking sgRNA or MLH1dn) into the original system to enhance the editing process [40]. Although many of these attempts have achieved remarkable progress, further research is still needed to optimize the technology. By exploring key factors that restrict the efficiency of PE editing, such as the process by which PE proteins interact with pegRNAs to exert editing functionality, their interplay with intracellular DNA repair pathways, and the optimization of PE protein and pegRNA sequences, the editing efficiency of PE editing can be further enhanced. In terms of precise editing, although the PE systems exhibit higher specificity than other editing systems, there is still a certain degree of off‐target risk. Therefore, the development of high‐fidelity prime editors is also crucial to ensuring the safety and accuracy of future therapies.

Additionally, limitations in the editing scope of PE systems must be considered. Although many genetic diseases are caused by single‐point mutations, there are numerous cases that are caused by multiple mutations or large insertions or deletions. Currently, PE is relatively effective in repairing point mutations and small insertions or deletions. However, mediating larger mutations or complex genomic rearrangements requires further development of the PE technology. Future efforts should aim to efficiently generate large DNA insertions and deletions by developing novel editing tools or combining existing strategies, thereby extending the application of PE editing to genetic disorders involving large fragment damage. An effective genome editing tool must possess a high level of precision in modifying the desired target and should minimize any unintended editing. The PE system utilizes Cas9 to cleave single‐stranded DNA. Simultaneously, the PE system necessitates the recognition of the targeted DNA strand along with the PBS and RTT regions of the pegRNAs during editing, leading to a reduced level of hybridization at the off‐target position. While these factors reduce the occurrence of off‐target effects, they do not completely eliminate them. This is particularly true for the PE3 and dual‐pegRNA systems, which are more prone to misediting when additional sgRNA is introduced to cleave the non‐edited strand or when dual pegRNAs are used to generate double‐strand breaks. Furthermore, the excessive expression of RT and Cas9 within the nucleus can result in the creation of undesired reverse transcriptional nuclear genome editing.

Previous research on base editing has demonstrated that off‐target editing takes place at genomic locations that share sequence similarity with the target site. This particular form of off‐targeting is referred to as sgRNA‐dependent off‐targeting. Aside from sgRNA‐dependent off‐targeting, there is another form of off‐target editing known as sgRNA‐independent off‐targeting. This occurs when deaminase domains on BEs bind to genomic DNA. At present, all off‐target editing in the PE process is caused by mutations that are dependent on pegRNAs. Gao et al. conducted a study that confirmed the absence of off‐target effects in both the entire genome and transcriptome. This was proved through an analysis of the off‐target effects of the PE3 system in a cell line with a low background mutation. They have shown that the off‐target effect caused by PE3, which does not depend on pegRNAs, was not observed at a comprehensive genomic and transcriptomic level, demonstrating the excellent specificity of the PE3 system [171].

To enhance the precision of PE editing, Anzalone et al. developed the PE3b system, which is an improvement upon the PE3 system. The PE3b system aims to minimize the occurrence of nicks on both DNA strands, thereby preventing the generation of unwanted byproducts while maintaining editing efficiency [23]. Jin et al. discovered that the off‐target effect of the PE system is influenced by the quantity and location of mismatches. This effect can be diminished by strategically constructing pegRNAs [172]. Transiently inhibiting the MMR pathway can enhance the efficiency and accuracy of PE as described above [40]. Additionally, the use of efficient delivery systems for gene editing tools, such as VLPs, can further improve editing efficiency and minimize off‐target effects [164]. When comparing plasmid transfection and viral transduction, the temporary transfer of prime editor proteins or mRNAs can reduce the exposure time of PE systems within cells, thereby decreasing off‐target effects.

Computational tools and methodologies are crucial in improving the performance of PE. These methods improve the process of pegRNA design and enhance the efficiency and precision of PE by evaluating substantial data or experimental outcomes, precisely creating pegRNAs, and predicting potential off‐target sites. Mathis et al. developed the machine learning model PRIDICT2.0/ePRIDICT to predict PE efficiency. The PRIDICT2.0 model excels in predicting editing efficiency by taking into account the diversity of chromatin environments. ePRIDICT predicts the editing efficiency of specific pegRNA and target site combinations. These two models are important for improving the efficiency of PE, especially when considering the complexity and diversity of chromatin states during genome editing [173]. Yu et al. have created various computational tools, including DeepPrime, DeepPrime‐FT, and DeepPrime‐Off, which can accurately forecast the editing effectiveness and unintended byproducts of eight PE systems across seven different cell types [174]. Liang et al. conducted a comprehensive analysis of PE data to forecast the efficiency of various PE systems and pegRNA combinations. As a result, they introduced a genome‐wide assay called PE‐tag, which is used to identify probable off‐target sites for PE [175]. Another technique for analyzing the genome editing capabilities of Cas9 is GUIDE‐seq [176]. This method can also be employed to evaluate off‐target locations for PE systems. Additionally, there are various alternative design and analysis tools and methods developed for PE editing, such as PrimeDesign [177], Easy‐Prime [178], and PEAC‐seq [179]. The advancement of these tools and approaches offers substantial assistance to scientists in optimizing PE based experiments, enhancing editing efficacy, minimizing off‐target effects, and facilitating the implementation of gene editing technology in the domains of medicine and biotechnology.

### 5.2 Efficient and Targeted Delivery of PE Systems

The development of efficient delivery vectors is crucial for the clinical application of PE. Due to its large size, delivering prime editors into the desired cells is challenging. Various delivery methods, such as physical strategies (e.g., electroporation and hydrodynamic injection) and viral vectors (e.g., AdVs and AAVs), have been explored to transfer the PE components of interest. However, safety concerns associated with these methods remain a major challenge for the broad application of gene editing in clinical trials. In recent years, non‐viral carriers (e.g., LNPs and VLPs) have emerged as promising alternatives and display high efficiency and low immunogenicity. For successful gene editing, precise targeting of desired cells in vivo is also of great importance. Therefore, the tissue and cell‐type specificity of delivery vectors must be carefully considered to improve the safety and efficacy of this precise gene editing tool. By utilizing different viral serotypes, modifying surface proteins, or employing tissue‐specific promoters, organ‐targeted delivery may be achieved via viral vectors/virus‐like particles. Moreover, incorporating targeting ligands into non‐viral carriers may be another promising approach to enhancing tissue and cell‐type specificity. With the emergence of more advanced targeted delivery technologies, we believe that the tremendous potential of PE in treating genetic diseases will be fully unlocked.

## 6 Conclusions

The design of novel gene editing tools has attracted global attention since the fast development of gene editing technology, which is considered the most promising approach to curing human genetic diseases. However, ethical and health concerns have emerged both in theory and in real‐life applications due to the potential of undesired edits at target and non‐target loci. The latest advancement in gene editing tools is PE, developed by Liu and co‐workers. PE stands out as the most efficient tool for precise editing, enabling single‐base substitutions and large fragment insertions or deletions, while generating minimum off‐target effects compared to other editing methods such as HDR‐mediated editing. Theoretically, PE holds the potential to efficiently and safely correct a wide range of human genetic disorders. This gene editing platform offers the bright prospect of unprecedented accuracy and efficiency in fundamental research and disease treatment.

In this review, we provide an overview of recent advancements in PE systems and current delivery strategies. Furthermore, we discuss current challenges and obstacles hindering clinical application, including low editing efficiency and fidelity, inefficient delivery, and a lack of tissue or cell type specificity. Despite the current challenges, the potential of PE in disease treatment and precision medicine is undeniable. Continued research and advancements in delivery technology, along with a comprehensive understanding of the key factors affecting editing efficiency, will undoubtedly unlock the full potential of PE in the treatment of genetic diseases in the future.

## Author Contributions

M.Y.L. wrote the manuscript. Y.L., Q.C., and T.W. reviewed and revised the manuscript. Q.C. and T.W. provided guidance throughout the preparation of the manuscript. All authors read and approved the final manuscript.

## Conflicts of Interest

The authors declare no conflicts of interest.

## Data Availability

Data sharing not applicable to this article as no datasets were generated or analysed during the current study.
